# Hypomorphic *Lig4* gene mutation in mice predisposes to Th1-skewed intestinal inflammation

**DOI:** 10.1172/jci.insight.199040

**Published:** 2026-09-01

**Authors:** Yusuke Yamashita, Hideki Kosako, Takashi Kato, Izumi Sasaki, Sadahiro Iwabuchi, Yuri Fukuda-Ohta, Tadashi Okamura, Misato Tane, Shotaro Tabata, Kazutaka Nakashima, Ken Tanaka, Kazunori Shiraishi, Yuki Uchihara, Daisuke Okuzaki, Kyoichi Isono, Atsushi Shibata, Tsunehiro Mizushima, Hiroaki Hemmi, Nobuo Kanazawa, Seiji Kodama, Hiroaki Miyoshi, Koichi Ohshima, Shinichi Hashimoto, Yoshio Fujitani, Takashi Sonoki, Shinobu Tamura, Tsuneyasu Kaisho

**Affiliations:** 1 Department of Hematology/Oncology,; 2 Department of Immunology, Institute of Advanced Medicine,; 3 Department of Rheumatology and Clinical Immunology, and; 4 Department of Molecular Pathophysiology, Institute of Advanced Medicine, Wakayama Medical University, Wakayama, Japan.; 5 Department of Pathology, Kurume University School of Medicine, Fukuoka, Japan.; 6 Laboratory of Radiation Biology, Department of Biological Chemistry, Graduate School of Science, Osaka Metropolitan University, Osaka, Japan.; 7 Division of Molecular Oncological Pharmacy, Faculty of Pharmacy, Keio University, Tokyo, Japan.; 8 Laboratory of Human Immunology (Single Cell Genomics), WPI Immunology Frontier Research Center, University of Osaka, Osaka, Japan.; 9 Laboratory Animal Center, School of Medicine, Wakayama Medical University, Wakayama, Japan.; 10 Graduate School of Science, University of Hyogo, Hyogo, Japan.; 11 Laboratory of Immunology, Faculty of Veterinary Medicine, Okayama University of Science, Ehime, Japan.; 12 Department of Dermatology, Hyogo Medical University, Hyogo, Japan.; 13 Laboratory of Developmental Biology and Metabolism, Institute for Molecular and Cellular Regulation, Gunma University, Gunma, Japan.; 14 First Department of Internal Medicine and; 15 Industry-Government-Academia Collaboration Promotion Headquarters, Wakayama Medical University, Wakayama, Japan.

**Keywords:** Immunology, Inflammation, Adaptive immunity, Inflammatory bowel disease, T cells

## Abstract

DNA ligase IV (LIGIV or LIG4) is essential for DNA double-strand break (DSB) repair. Hypomorphic *LIG4* variants cause LIG4 syndrome, characterized by growth disturbance, increased radiosensitivity, predisposition to malignancies, adaptive immunodeficiency, and inflammatory conditions. Most of these manifestations are recapitulated in hypomorphic *Lig4* mutant mice. However, no model mice with defective DSB repair have consistently exhibited inflammation. Here, we generated mutant mice carrying the *LIG4* missense variant, p.W447C, found in a patient with LIG4 syndrome. *Lig4^W447C/W447C^* mice showed functional defects of LIGIV and manifested growth retardation, increased radiosensitivity, and life-threatening intestinal inflammation under severe adaptive immunodeficiency. The inflammation was dependent on lymphocytes and characterized by marked infiltration of Th1 cells and macrophages, along with elevated expression of IFN-γ–inducible genes. When *Ifng* was deleted, Th2 and Th17 instead of Th1 cells drove the inflammation. Single-cell RNA-seq analyses with TCR repertoire revealed that T cells from *Lig4^W447C/W447C^* mice preferentially used proximal Vα and Jα segments in V regions of TCRα chains and exhibited expansion of several clonotypes, a substantial portion of which were CD4^+^ T cells expressing IFN-γ. Thus, our hypomorphic *Lig4* mutant mice represent a unique model for studying Th1-skewed intestinal inflammation under severe adaptive immunodeficiency.

## Abstract 2

A partial defect in DNA repair weakens immune defenses yet promotes severe intestinal inflammation in mice, linking faulty DNA repair to abnormal immune responses.

## Graphical abstract

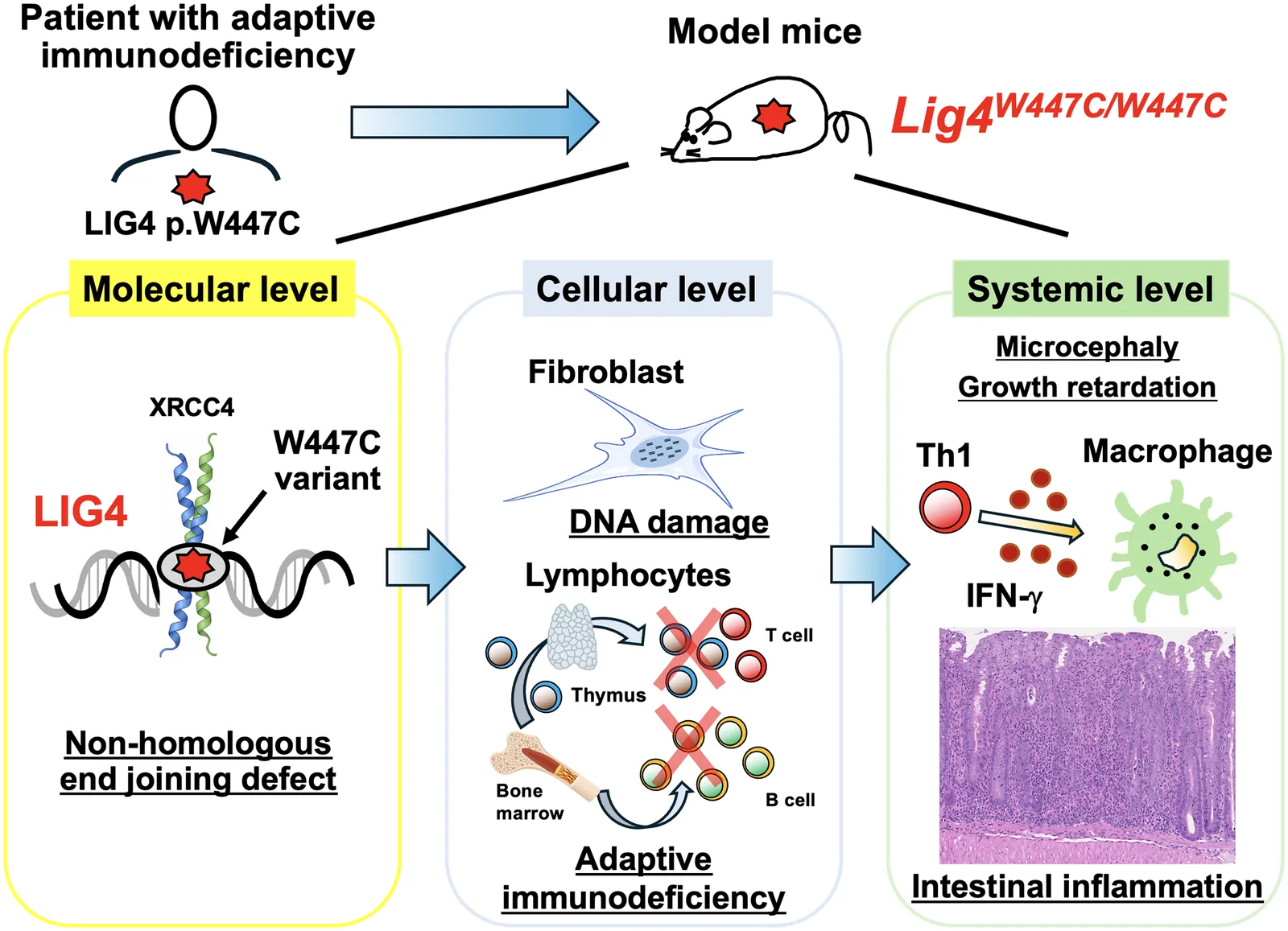

## Introduction

In mammals, DNAs are damaged to generate DNA double-strand breaks (DSBs) not only upon exposure to ionizing radiation or ultraviolet, but also in the physiological development of nervous and immune systems. Non-homologous end joining (NHEJ) is a critical pathway to repair DSBs (1–3). In lymphocytes, NHEJ is indispensable for productive V(D)J recombination of T cell receptor (TCR) and Ig genes. NHEJ is mediated by a series of reactions, in which there is involvement of DNA-dependent protein kinase catalytic subunit (DNA-PKcs), the KU70/80 heterodimer, Artemis, Cernunnos (also known as XRCC4-like factor [XLF]), DNA ligase IV (LIGIV), and X-ray repair cross-complementing protein 4 (XRCC4) (1–3). In humans, defects in any of these components can lead to profound adaptive immunodeficiency by impairment of the V(D)J recombination, increased radiosensitivity, and neurodevelopmental delay (4).

LIGIV binds to DSB ends and acts as the ligase with XRCC4 in the final end-joining step of NHEJ (5, 6). Hypomorphic variants in the human *LIG4* gene underlie LIG4 syndrome, a rare autosomal recessive disorder characterized by growth disturbance, neurodevelopmental delay, increased radiosensitivity, and adaptive immunodeficiency (5–7). Patients with LIG4 syndrome have also shown a predisposition to malignancies including leukemia and lymphoma (8–11). Paradoxically, because of dysregulated activation of adaptive immunity, they occasionally exhibit inflammatory disorders such as inflammatory bowel disease and Behçet diseases (12–14). Although LIG4 syndrome exhibits phenotypic heterogeneity, little is known about how the variants of the human *LIG4* gene contribute to diverse manifestations such as inflammatory conditions.

In mice, *Lig4* deficiency causes embryonic lethality due to impaired neuronal development (15, 16). Two kinds of mutant mice carrying a hypomorphic *Lig4* variant in the enzymatic domain have been generated to date, and homozygous mutant mice showed neurodevelopmental delay from birth as well as adaptive immunodeficiency (17–21). In mice homozygous for a *Lig4* R278H mutation (*Lig4^R278H/R278H^*) derived from a patient with LIG4 syndrome, thymic T cell lymphoma was observed at high frequencies (19). Moreover, *Lig4^Y288C/Y288C^* mice, which were generated by *N*-ethyl-*N*-nitrosourea, also exhibited a high incidence of lymphoid neoplasms that originate from the thymus (20). LIG4 syndrome manifestations were thus well recapitulated in the mutant mice. However, none of these mutant mice show any apparent signs of inflammation.

We have previously described a patient with LIG4 syndrome who was carrying a compound heterozygous variant in the *LIG4* gene. One variant was a nonsense variant, p.E413X; the other one was a missense variant, p.W447C, found in the well-conserved amino acid among species (22). In the current study, we generated mutant mice carrying this missense variant. *Lig4^W447C/W447C^* mice manifested not only neuronal and developmental defects, but also lymphocyte-dependent colitis with adaptive immunodeficiency. The colitis was characterized by infiltration of Th1 cells and macrophages and lack of B cells. *Lig4^W447C/W447C^* mice are unique immunodeficient mice manifesting Th1 cell–driven inflammatory disorders.

## Results

### Lig4^W447C/W447C^ mice show microcephaly, growth retardation, and radiosensitivity.

To clarify the pathological roles of a missense variant (p.W447C), we introduced the variant in mice by the CRISPR/Cas9 system. *Lig4^W447C/+^* mice were born and appeared healthy. After crossing between *Lig4^W447C/+^* mice, *Lig4^W447C/W447C^* mice were born with expected Mendelian inheritance but showed short stature and low birth weight (Supplemental Figure 1A; supplemental material available online with this article; https://doi.org/10.1172/jci.insight.199040DS1). The mice also exhibited growth retardation and microcephaly (Figure 1A and Supplemental Figure 1B). These manifestations are hallmarks of the LIG4 syndrome (5–7). We then investigated DSBs by formation of γH2AX foci in mouse embryonic fibroblasts (MEFs). The formation was comparable between *Lig4^+/+^* and *Lig4^W447C/+^* MEFs. In *Lig4^W447C/W447C^* MEFs, more γH2AX foci were detected not only before but also upon irradiation than in *Lig4^W447C/+^* MEFs (Figure 1B). Thus, *Lig4^W447C/W447C^* mice showed growth retardation, microcephaly, and defects in DNA damage repair.

Expression of LIGIV protein in *Lig4^W447C/W447C^* MEFs was at comparable levels to that in wild-type MEFs, indicating that the mutation does not affect the amounts of LIGIV protein (Figure 1C). In addition, the expression levels of XRCC4 and XLF, which assist LIGIV in its DNA repair function, were also unchanged between *Lig4^W447C/W447C^* and wild-type MEFs (Figure 1C). We therefore performed in silico analysis on wild-type and the W447C mutant of LIGIV. W447 was localized en face to AMP in the enzymatic domain. The W447C mutant did not affect overall structures, but it resulted in a decrease of the AMP-binding interface area (wild-type 2.66 Å^2^, W447C mutant 1.98 Å^2^) and enlargement of the AMP-binding pocket mainly due to lack of an indole ring (Figure 1, D and E) (23). We also analyzed the R278H and Y288C mutants of LIGIV (Supplemental Figure 1, C and D), but neither mutant altered overall structures of LIGIV. Y288 did not face to AMP, while R278 was in contact with AMP and the R278H mutant decreased the interface area (wild-type 27.84 Å^2^, R278H mutant 18.46 Å^2^) but did not significantly affect the structure of the AMP-binding pocket.

We next investigated functional consequences of W447C mutation through the in vitro plasmid-based DNA end-joining assay. *Lig4^+/+^* MEF extracts exhibited robust DNA end-joining activity (31.9% ± 11.3%), whereas *Lig4^W447C/W447C^* MEF extracts showed markedly reduced activity (1.04% ± 0.2%) (Figure 1F). The results indicate that the *Lig4* W447C mutation severely impairs the LIGIV-dependent final ligation step of NHEJ.

### Lig4^W447C/W447C^ mice exhibit severe adaptive immunodeficiency.

We then analyzed the phenotype of lymphocytes in *Lig4^+/+^* and *Lig4^W447C/W447C^* mice. *Lig4^W447C/W447C^* mice had half the number of BM cells of *Lig4^+/+^* mice (Figure 2, A and B). In the BM of *Lig4^W447C/W447C^* mice, B220^+^CD43^+^IgM^−^ pro–B cells were decreased, and there was marked reduction in B220^+^CD43^−^IgM^−^ pre–B cells, B220^lo^CD43^−^IgM^+^ immature B cells, and B220^hi^CD43^−^IgM^+^ mature B cells (Figure 2, C and D). Meanwhile, the proportion of pro–B cells among B220^+^ cells was significantly increased, indicating incomplete block at the pro–B cell stage, which was consistent with impaired rearrangement of the IgH locus (Figure 2, C and D). In the spleens of *Lig4^W447C/W447C^* mice, follicular formation was severely impaired, and the numbers of splenocytes and B220^+^ cells were severely decreased (Figure 2, E–H). Furthermore, in *Lig4^W447C/W447C^* mice, serum IgM, IgG1, and IgA levels were severely decreased (Figure 2I). Notably, serum IgE levels remained low, similar to those of *Lig4^+/+^* mice (Figure 2I). In addition, the serum levels of anti-dsDNA antibody in *Lig4^W447C/W447C^* mice were lower than those in *Lig4^+/+^* mice (Supplemental Figure 2A). Thus, B cell generation was defective in *Lig4^W447C/W447C^* mice.

In *Lig4^W447C/W447C^* mice, the thymus was very small (Figure 2J), and histological analysis showed unclear corticomedullary boundary (Figure 2K). The proportion of CD3^+^ cells in thymocytes of *Lig4^W447C/W447C^* mice was severely decreased compared with that of *Lig4^+/+^* mice (1.7% vs. 19%) (Figure 2L). Furthermore, there were very few T-lineage cells, such as double-negative (DN) CD4^−^CD8^−^, double-positive (DP) CD4^+^CD8^+^, and single-positive (SP) CD4^+^ or CD8^+^ thymocytes (Figure 2M). The percentages of DN thymocytes in the *Lig4^W447C/W447C^* mice were increased, whereas those of DP and SP CD4^+^ thymocytes were decreased (Figure 2L and Supplemental Figure 2B). In the spleen, CD3^+^CD4^+^ and CD3^+^CD8^+^ T cells were severely decreased in *Lig4^W447C/W447C^* mice (Figure 2, G and N–P). Notably, the CD4/CD8 ratio was higher in *Lig4^W447C/W447C^* mice than in *Lig4^+/+^* mice (Figure 2Q). The majority of CD4^+^ and CD8^+^ T cells remaining in the mutant mice were effector memory T cells (CD44^hi^CD62L^lo^), and naive T cells (CD44^lo^CD62L^hi^) were hardly detected (Figure 2, R and S). Concerning splenic Foxp3^+^ Tregs in CD4^+^ T cells, the percentages were lower in *Lig4^W447C/W447C^* than in *Lig4^+/+^* mice (Supplemental Figure 2, C and D). Taken together, *Lig4^W447C/W447C^* mice showed severe defects in both B and T cell generation, i.e., severe adaptive immunodeficiency.

### Lig4^W447C/W447C^ mice develop severe intestinal inflammation.

After weaning, *Lig4^W447C/W447C^* mice exhibited varying degrees of wasting, diarrhea, and rectal prolapse and showed higher mortality than *Lig4^+/+^* and *Lig4^W447C/+^* mice when housed in specific pathogen–free conditions. Kaplan-Meier analysis revealed that *Lig4^W447C/W447C^* mice had a median overall survival of 92 days (Figure 3A). In the macro-anatomical study, marked edema and shortening of the large intestine were found in almost all *Lig4^W447C/W447C^* mice around 10 weeks of age (Figure 3B). Histological examination of the large intestines revealed epithelial hyperplasia, goblet cell disappearance, and infiltration of inflammatory cells in the lamina propria and submucosa in *Lig4^W447C/W447C^* mice (Figure 3C). Mouse colitis histology index (MCHI) of the *Lig4^W447C/W447C^* mice was significantly higher than that of *Lig4^+/+^* mice (Figure 3D). Moreover, crypt abscesses, which are formed by active inflammation and are a hallmark of ulcerative colitis, were often present in the *Lig4^W447C/W447C^* mice (Figure 3C, arrowhead). Immunohistochemical analysis of the colons further showed that infiltrated cells in *Lig4^W447C/W447C^* mice consisted mainly of T cells, especially CD4^+^ T cells, and F4/80^+^ macrophages (Figure 3E). These pathological findings were also observed in the small intestines of *Lig4^W447C/W447C^* mice (Supplemental Figure 3, A and B). In *Lig4^W447C/W447C^* mice, increased T cells were mainly TCRαβ^+^ cells rather than TCRγδ^+^ cells, although their ratio of CD4 to CD8 was similar to that of T cells in *Lig4^+/+^* mice (Supplemental Figure 3, C and D). Meanwhile, B220^+^ cells were hardly detected (Figure 3E and Supplemental Figure 3A). *Lig4^W447C/W447C^* mice therefore showed severe inflammation in the small and large intestines with infiltration mainly of CD4^+^ T cells and macrophages into the mucosa and submucosa.

### Intestinal inflammation in Lig4^W447C/W447C^ mice is dependent on lymphocytes.

Next, to determine whether hematopoietic cells were sufficient for the development of colitis in the *Lig4^W447C/W447C^* mice, we generated BM chimeric mice by transferring BM cells from *Lig4^+/+^* and *Lig4^W447C/W447C^* mice into irradiated *Rag2^−/−^* mice, which are hereafter referred to as *Lig4^+/+^*→*Rag2^−/−^* and *Lig4^W447C/W447C^*→*Rag2^−/−^* mice, respectively (Figure 3F). Compared with *Lig4^+/+^*→*Rag2^−/−^* mice, *Lig4^W447C/W447C^*→*Rag2^−/−^* mice showed severe intestinal manifestations, including diarrhea, colonic shortness, and edema (Figure 3G). Furthermore, in the colons of *Lig4^W447C/W447C^*→*Rag2^−/−^* mice, mainly CD4^+^ T cells and macrophages infiltrated, as observed in the colons of *Lig4^W447C/W447C^* mice (Figure 3, H and I). These results suggest that hematopoietic cells are responsible for the development of intestinal inflammation in *Lig4^W447C/W447C^* mice. Meanwhile, *Lig4^W447C/W447C^* mice were sensitive to and died from irradiation. Therefore, we could not generate BM chimeric mice by using *Lig4^W447C/W447C^* mice as recipients. Thus, we could not assess the roles of non-hematopoietic cells in intestinal inflammation in *Lig4^W447C/W447C^* mice.

To further investigate the involvement of lymphocytes in intestinal inflammation in the *Lig4^W447C/W447C^* mice, *Lig4^W447C/W447C^ Rag2^−/−^* mice were generated by crossing of *Lig4^W447C/W447C^* mice with *Rag2^−/−^* mice. Splenic T cells were still detected in *Lig4^W447C/W447C^* mice, but they were absent in *Lig4^W447C/W447C^ Rag2^−/−^* mice (Figure 3J). The double-mutant mice had growth disturbances and microcephaly but showed significantly longer survival than *Lig4^W447C/W447C^* mice (Figure 3, K and L, and Supplemental Figure 4). Notably, *Lig4^W447C/W447C^ Rag2^−/−^* mice developed no signs of intestinal inflammation (Figure 3, M and N). These results suggest that lymphocytes are required for the development of intestinal inflammation in *Lig4^W447C/W447C^* mice.

Innate lymphoid cells (ILCs) are innate immune cells that lack antigen-specific receptors and are classified into ILC1, ILC2, and ILC3 subsets, which produce cytokines similar to those produced by Th1, Th2, and Th17 cells, respectively (24). These subsets can contribute to various inflammatory conditions, including colitis. We therefore analyzed ILC subsets using the lineage-defining transcription factors T-bet, GATA3, and RORγt, which include ILC1, ILC2, and ILC3, respectively. In *Lig4^W447C/W447C^* mice, the percentages of T-bet^+^ and RORγt^+^ cells as well as the expression levels of these transcription factors were reduced (Supplemental Figure 5, A and B).

### Expression of IFN-γ–inducible genes is enhanced in the colon of Lig4^W447C/W447C^ mice.

To characterize intestinal inflammation in *Lig4^W447C/W447C^* mice, RNA sequencing (RNA-seq) analysis was performed on colonic tissues from *Lig4^+/+^* and *Lig4^W447C/W447C^* mice. In total, 25,706 genes were differentially expressed between *Lig4^+/+^* and *Lig4^W447C/W447C^* mice (Supplemental Table 1). Expression of 2,278 and 3,728 genes was up- and downregulated, respectively, more than 1.5-fold. Unbiased gene set enrichment analysis (GSEA) of these genes revealed up- and downregulation of 24 and 2 gene sets, respectively (nominal *P* value < 0.05, false discovery rate *q* value < 0.25) (Figure 4A and Supplemental Table 2). The most upregulated gene set was “interferon-gamma response” (normalized enrichment score = 2.97; *P* < 0.001) (Figure 4, A–C). Expression of 15 representative IFN-γ–inducible genes such as *Ciita* or *Cxcl9* was more than 5-fold higher in the colons of *Lig4^W447C/W447C^* mice than in those of *Lig4^+/+^* mice (Figure 4B and Supplemental Table 1). Furthermore, expression of these genes was more prominently enhanced in the colons from *Lig4^W447C/W447C^* mice with severe manifestations (MCHI: #3, score 17; #4, score 20) versus relatively mild manifestations (MCHI: #1, score 9; #2, score 3) (Figure 4D). Thus, intestinal inflammation in *Lig4^W447C/W447C^* mice is characterized by enhanced production of IFN-γ.

### Loss of IFN-γ expression in Lig4^W447C/W447C^ mice evokes Th2/Th17-skewed intestinal inflammation.

IFN-γ is a key cytokine expressed in Th1 cells among lymphocytes. We therefore compared the expression of various Th cell subset signature genes. Th1 cell signature genes including *Ifng* were expressed at significantly higher levels in *Lig4^W447C/W447C^* colons than in *Lig4^+/+^* colons (Figure 5A). Expression of Th2 cell and Treg signature genes was also increased, whereas expression of some Th17 cell signature genes was rather decreased, in the mutant colons (Figure 5A).

We also performed intracellular cytokine staining analysis in CD4^+^ T cells from *Lig4^+/+^* and *Lig4^W447C/W447C^* mice. IFN-γ–expressing CD4^+^ T cells were higher in frequency in the spleen, mesenteric lymph nodes (MLNs), lamina propria (LP), and intraepithelial compartment of *Lig4^W447C/W447C^* mice than in those of *Lig4^+/+^* mice (Figure 5, B and C, and Supplemental Figure 6, A and B). Notably, almost half of splenic CD4^+^ T cells expressed IFN-γ in *Lig4^W447C/W447C^* mice. Meanwhile, IL-4–expressing CD4^+^ T cells were comparable (Figure 5B). The frequency of IL-17A–expressing CD4^+^ T cells was increased among MLN lymphocytes, LP lymphocytes, and intraepithelial lymphocytes in *Lig4^W447C/W447C^* mice compared with wild-type mice, although it remained below 10% (Figure 5B and Supplemental Figure 6A). Among LP CD4^+^ T cells, IL-17A–expressing cells were dominant in *Lig4^+/+^* mice, whereas IFN-γ–expressing cells were dominant in the mutant mice (Figure 5D). Dominance of IFN-γ–expressing cells was more prominent in the spleen and MLN CD4^+^ T cells in *Lig4^W447C/W447C^* mice. These results suggest that Th1 cells producing IFN-γ are mainly activated in *Lig4^W447C/W447C^* mice.

To examine whether altered thymic egress might be associated with the Th1-skewed responses in *Lig4^W447C/W447C^* mice, we examined thymic egress by analyzing expression of thymic egress markers in SP CD4^+^ thymocytes. SP CD4^+^ thymocytes mature from CD69^+^MHC class I^–^ semimature (SM) through CD69^+^MHC class I^+^ mature 1 (M1) to CD69^–^MHC class I^+^ mature 2 (M2) cells, which also express Qa-2 and S1PR1 (25). Compared with those from *Lig4^+/+^* mice, most SP CD4^+^ cells were CD69^–^MHC class I^+^S1PR1^–^Qa-2^+^ in *Lig4^W447C/W447C^* mice (Supplemental Figure 7). Notably, the expression levels of MHC class I and Qa-2 in CD69^–^ SP CD4^+^ cells were higher in *Lig4^W447C/W447C^* mice than in *Lig4^+/+^* mice. Considering that Qa-2 and MHC class I are upregulated during thymocyte maturation and are highly expressed in mature peripheral T cells (26), the results suggest that SP CD4^+^ cells from *Lig4^W447C/W447C^* mice may represent recirculating peripheral T cells rather than developing thymocytes.

To determine whether and how IFN-γ is involved in the development of intestinal inflammation in *Lig4^W447C/W447C^* mice, we generated *Lig4^W447C/W447C^ Ifng^−/−^* mice. The double-mutant mice showed developmental defect and microcephaly, similar to *Lig4^W447C/W447C^* mice. The double-mutant mice had inflammatory cell infiltration in the intestine, with a similar inflammatory score to that of the *Lig4^W447C/W447C^* mice (Figure 5, E and F). Although crypt abscesses were not observed, neutrophils were more markedly increased, while macrophages (F4/80^+^ cells) were increased to a comparable extent, relative to *Lig4^W447C/W447C^* mice (Figure 5, E and G). Furthermore, in the double-mutant mice, eosinophils tended to increase (Figure 5E, dashed circle, and Figure 5G), and multinucleated giant cells, which could be potentially derived from activated macrophages, were occasionally found (Figure 5E, arrow, and Figure 5G). Meanwhile, mast cells were hardly detected in both *Lig4^W447C/W447C^* and *Lig4^W447C/W447C^ Ifng^−/−^* mice. Flow cytometric analysis revealed residual T cells in the splenocytes of *Lig4^W447C/W447C^ Ifng^−/−^* mice (Figure 5H). Intracellular cytokine staining analysis revealed that the residual splenic T cells showed no IFN-γ expression but increased IL-4 and IL-17A expression (Supplemental Figure 8). IL-17A–expressing T cells were also increased in the LP and intraepithelial compartment of *Lig4^W447C/W447C^ Ifng^−/−^* mice (Figure 5, I and J, and Supplemental Figure 9, A and B). The transcriptome analysis also revealed that expression of IFN-γ–inducible genes was profoundly decreased in the colons of *Lig4^W447C/W447C^ Ifng^−/−^* mice compared with *Lig4^W447C/W447C^* mice (Figure 5, K and L, and Supplemental Table 1). GSEA comparing *Lig4^W447C/W447C^ Ifng^−/−^* and *Lig4^W447C/W447C^* mice showed downregulation and upregulation of 18 and 5 gene sets, respectively (nominal *P* value < 0.05, false discovery rate *q* value < 0.25), and confirmed that the most downregulated gene set was “interferon-gamma response” (Figure 5, M and N, and Supplemental Table 2). Furthermore, we compared the expression of various Th cell subset signature genes. Expression of Th1 cell signature genes in the *Lig4^W447C/W447C^ Ifng^−/−^* colons was returned to comparable levels to those in the *Lig4^+/+^ Ifng^+/+^* colons (Supplemental Table 1). Meanwhile, expression of Th17 (*Il17a* and *Cxcl1*) and Th2 (*Ccl17* and *Ccl24*) cell signature genes was upregulated in *Lig4^W447C/W447C^ Ifng^−/−^* colons compared with *Lig4^+/+^ Ifng^+/+^* and *Lig4^W447C/W447C^ Ifng^+/+^* colons (Figure 5, O and P, and Supplemental Table 1). Expression of Treg signature genes was comparable among these mice (Figure 5O and Supplemental Table 1). Taken together, IFN-γ deficiency ameliorated Th1-skewed intestinal inflammation but instead deteriorated Th2/Th17-skewed intestinal inflammation in *Lig4^W447C/W447C^* mice.

### Lig4^W447C/W447C^ mice exhibit a biased TCR repertoire in the spleen.

We then performed a comprehensive bulk-based analysis of a diverse repertoire of T cell receptors in the spleen and the MLN to investigate whether T cell repertoire shows some skewing in *Lig4^W447C/W447C^* mice. The T cell repertoire was quite heterogeneous in *Lig4^+/+^* mice, while certain T cell receptors were abundantly expressed in *Lig4^W447C/W447C^* mice (Supplemental Figure 10, A–D, and Supplemental Tables 3 and 4). A close examination of the 3′ (proximal) Vα segments revealed a striking difference in their usages among *Lig4^+/+^* and *Lig4^W447C/W447C^* mice (Supplemental Figure 10, A and B). TCR Vα usage in the spleen and the MLN of *Lig4^W447C/W447C^* mice was strongly skewed to the 3′ (proximal) end. Meanwhile, as for Vβ, a slight bias in the utilization frequency of segments was observed in *Lig4^W447C/W447C^* mice, regardless of their position (Supplemental Figure 10, C and D).

Next, we performed single-cell RNA-seq (scRNA-seq) analysis to investigate which pairs of T cell receptors are expressed in the spleens of *Lig4^+/+^* and *Lig4^W447C/W447C^* mice. Consistent with flow cytometry analysis, T cells were severely decreased, and the residual T cells mainly consisted of Th1 cells in the spleens of the mutant mice (Figure 6, A and B, Supplemental Figure 11, and Supplemental Table 5). *Lig4^+/+^* T cells from 2,015 barcodes included 2,002 clonotypes, which contained the clones limited to two at most, and all but the top 6 clonotypes were represented by one cell. Meanwhile, 667 *Lig4^W447C/W447C^* T cells consisted of 235 clonotypes, and the top 10 clonotypes each occupied more than 10 T cells, 1.5% of the total T cells (experiment 1 in Figure 6C). Those 10 clones contained 4 CD4^+^ cells, 5 CD8^+^ cells, and 1 CD4^−^CD8^−^ T cell. Seven of 10 clones did not express *Sell*, indicating that most of them were activated. Two CD4^+^ T cell clonotypes, which were activated and expressed *Trbv3*/*Trbj1-1* or *Trbv16*/*Trbj2-3*, showed high expression of *Ifng* (experiment 1 in Figure 6, D and E). Meanwhile, neither of these 10 T cell clonotypes expressed *Il4* or *Il17a* (experiment 1 in Figure 6D). Furthermore, we analyzed the T cell repertoire diversity in 2 additional *Lig4^W447C/W447C^* spleens (experiments 2 and 3) by scRNA-seq analysis. In experiment 2 and 3 mutant mice, 1,265 and 1,610 T cells consisted of 366 and 416 clonotypes, respectively (Figure 6C). These top 10 clonotypes each occupied 1.26% and 1.30% of the total T cells (experiments 2 and 3 in Figure 6C). In mutant mouse #2, two CD4^+^ T cell clonotypes expressing *Ifng* represented *Trbv3*/*Trbj2-3*/*Trav14-3*/*Traj56* or *Trbv15*/*Trbj2-5*/*Trav13-1*/*Traj58* transcripts (experiment 2 in Figure 6, D and E). Meanwhile, in mutant mouse #3, all the top 9 clonotypes were effector memory CD4^+^ T cells. However, none of them expressed *Ifng*, *Il4*, or *Il17a* (experiment 3 in Figure 6, D and E). Therefore, several T cell clonotypes, which may include clonotypes with pathogenic potential, were skewed and expanded in the mutant mice.

Moreover, we quantitated usage of TCR V and J gene segments (Vα, Jα, Vβ, and Jβ) in CDR3 sequences by scRNA-seq analysis in splenic T cells of one *Lig4^+/+^* and three *Lig4^W447C/W447C^* mice (Figure 7, A–F). The Vα repertoire in T cells from 3 mutant mice was dominated by the most 3′ (proximal) segments from *Trav16* to *Trav21-dv12* segments (Figure 7, A and E). Conversely, the Jα repertoire in the mutant T cells was dominated by the most 5′ (proximal) segments from *Traj58* to *Traj49* (Figure 7, B and E). Moreover, Vβ and Jβ segments in the mutant T cells were evenly distributed, although usage of some segments, including *Trbv1*, *Trbv3*, and *Trbj1-1*, was increased (Figure 7, C, D, and F).

We next analyzed TCR Vα and Vβ segment usage in CD4^+^ T cells (Supplemental Figure 12, A–C) and examined expression of selected Vα and Vβ chains by flow cytometry (Supplemental Figure 12D). In mutant mice, Vα2^+^ T cells, which express the TCR Vα chain encoded by the proximal segments, the *Trav14* family, were significantly increased. Vβ11 (*Trbv16*)^+^ T cells also tended to increase, whereas Vβ8 (*Trbv13* family)^+^ T cells were comparable to those in wild-type mice. These flow cytometric data were consistent with the scRNA-seq results (Supplemental Figure 12).

## Discussion

We introduced a novel hypomorphic gene variant, p.W447C, derived from a patient with LIG4 syndrome into mice. The homozygous mutant mice showed not only developmental and neuronal defects but also severe adaptive immunodeficiency. They further showed severe intestinal inflammation with intestinal epithelial hyperplasia, marked infiltration of Th cells and macrophages, and crypt abscesses, which are characteristic of ulcerative colitis. The inflammation was developed in *Lig4^W447C/W447C^*→*Rag2^−/−^* mice and abolished in *Lig4^W447C/W447C^ Rag2^−/−^* mice, indicating critical involvement of hematopoietic cells, especially lymphocytes, in intestinal inflammation. Furthermore, the lesions showed high expression of IFN-γ and IFN-γ–induced genes and were skewed toward Th2/Th17-type inflammation by IFN-γ deficiency. These results indicate that intestinal inflammation in *Lig4^W447C/W447C^* mice is driven by Th1 cells.

LIGIV-deficient mice are embryonic lethal. Meanwhile, so far, 2 kinds of hypomorphic *Lig4* homozygous mutant mice, Y288C and R278H, have been generated and were found to be born with growth retardation (16–21). Both *Lig4^Y288C/Y288C^* and *Lig4^R278H/R278H^* mice manifested defective DSB repair and adaptive immunodeficiency, as observed also in *Lig4^W447C/W447C^* mice (17, 19, 20). Although protein expression of LIGIV is decreased in *Lig4^Y288C/Y288C^* and *Lig4^R278H/R278H^* mice, it is preserved in *Lig4^W447C/W447C^* mice (19, 21). This indicates that protein stability or synthesis is impaired by Y288C and R278H, but not by W447C mutations, and it indicates that defective protein expression caused by Y288C and R278H mutations should contribute to functional defects of LIGIV. Although the W447C LIGIV protein was expressed, in vitro DNA end-joining assay using the cell extracts from *Lig4^+/+^* and *Lig4^W447C/W447C^* mice revealed a marked functional defect in the mutant protein.

It remains unknown why W447C mutation, which does not reduce LIGIV expression, caused more severe adaptive immunodeficiency than the 2 other mutations. Y288, R278, and W447 are all localized in the middle of the enzymatic domain. According to in silico analysis, neither Y288C, R278H, nor W447C mutations disturb the overall structure of LIGIV. In order to repair DSB, LIGIV should first bind to AMP (23). W447 faces to AMP, and W447C mutation is supposed to cause decrease of the interface area and enlargement of the AMP-binding pocket, which should then lead to destabilization of the interaction of LIGIV and AMP. Although R278 also faces to AMP and R278H mutation leads to decrease of the interface area, the mutation minimally affects the AMP-binding pocket. Y288 is located on the surface of LIGIV and is not directly involved in the interaction of LIGIV with AMP. The AMP-binding pocket should therefore be structurally kept by Y288C mutation. Based on these findings, we speculate that W447C mutation may disrupt the interaction of LIGIV with AMP more severely than the other 2 mutations, resulting in a greater reduction in enzymatic activity and thereby leading to more severe defects in adaptive immunity. However, since the 3 mutations have not been directly compared, it remains unclear whether this is indeed the case.

Among 3 hypomorphic *Lig4* homozygous mutants, *Lig4^W447C/W447C^* mice are characterized by their inflammatory phenotype. Meanwhile, *Lig4^R278H/R278H^* and *Lig4^Y288C/Y288C^* mice showed increased morbidity and high incidence of thymic tumors, indicating that these 2 mutations led to increased susceptibility to malignancy (19, 20). In this study, we could not formally assess whether *Lig4^W447C/W447C^* mice manifest the lymphoid malignancies, because most of them do not survive long enough to be analyzed. No apparent inflammatory conditions have been found in these previously reported mice, although only a modest infiltration of T lymphocytes and neutrophils was observed in the guts and livers of *Lig4^R278H/R278H^* mice (17, 19). Furthermore, splenic T cells in the *Lig4^R278H/R278H^* mice did not show any prominent skewing in their cytokine expression profiles (19). Thus, Th1 cell–driven intestinal inflammation with macrophages in the *Lig4^W447C/W447C^* mice is a unique phenotype. At present, however, we cannot exclude the possibility that the difference in susceptibility to inflammation among these *Lig4* mutant mice is caused by certain environmental factors. It is especially important to determine whether the intestinal microbiota contributes to development of inflammation and, if so, to identify the specific microbial species involved. The potential contribution of the intestinal microbiota therefore warrants further investigation, including longitudinal metagenomics, reciprocal microbiota transfer, and gnotobiotic colonization.

The RAG proteins, involved in V(D)J recombination of B cell and T cell receptors, are essential for lymphocyte development. Several hypomorphic mutations in *Rag* genes also lead to inflammatory manifestations driven by lymphocytes with adaptive immunodeficiency, which are called Omenn syndrome (27–31). Among several *Rag* mutant mice, *Rag2^R229Q/R229Q^* and *Rag1^R972W/R972W^* mice have manifested inflammatory conditions, including colitis, which are similar to intestinal inflammation in *Lig4^W447C/W447C^* mice in terms of dominant infiltration of Th cells (32, 33). However, *Rag2^R229Q/R229Q^* mice show not only Th1- but also Th17- and Th2-driven inflammation, which is characterized by skin lesions with erythroderma and eosinophilia (33–35). In *Rag1^R972W/R972W^* mice, systemic and colonic Th1 and Th17 inflammatory signatures are observed (32). Meanwhile, in *Lig4^W447C/W447C^* mice, the intestinal inflammation was driven mainly by Th1 cells, and skin lesions were not observed. Furthermore, in *Lig4^W447C/W447C^* mice, B-lineage cells were hardly detected in the intestines or spleen, and serum Ig levels including IgE and anti-dsDNA antibodies were low. This is in contrast to the findings on *Rag2^R229Q/R229Q^* and *Rag1^R972W/R972W^* mice, showing that levels of serum IgM and IgGs were preserved and that levels of serum IgE and anti-dsDNA Abs were increased (34, 36). Thus, inflammation in *Lig4^W447C/W447C^* mice is pathologically distinct from that in *Rag2^R229Q/R229Q^* and *Rag1^R972W/R972W^* mice. The difference should come from broader expression of LIGIV than RAG. Non-lymphocytes with defective LIGIV, which are prone to DSB-induced effects such as cell death, might be involved in generation of inflammation.

RAG deficiency in *Lig4^W447C/W447C^* mice led to the disappearance of intestinal inflammation (Figure 3), suggesting that T cells play an essential role in disease development, whereas ILCs may not be strictly required. In *Lig4^W447C/W447C^* mice, the proportions of T-bet^+^ and RORγt^+^ cells, as well as the expression levels of these transcription factors, were reduced (Supplemental Figure 5). At present, the significance of the altered expression of these markers remains to be determined. Decreased RORγt expression might be a consequence of intestinal inflammation, given that a similar decrease in ILCs has been reported under certain inflammatory conditions (37). Nevertheless, whether the observed changes contribute to or result from intestinal inflammation remains unclear. The potential contribution of *Lig4^W447C/W447C^* intestinal epithelial or stem cells to colitis development has also not been formally examined. Notably, LIGIV has been implicated in Wnt signaling–induced radioresistance of intestinal stem cells (38), suggesting a potential contribution of these cells to colitis. Although the absence of intestinal inflammation in RAG-deficient *Lig4^W447C/W447C^* mice indicates that *Lig4^W447C/W447C^* non-lymphoid cells alone are insufficient to induce colitis, a contribution of the mutant intestinal stem cells to colitis development cannot be fully excluded. Thus, further investigation is required to clarify the potential involvement of non-lymphocytes in disease pathogenesis.

Characterization of CD4^+^ TCR clonality has been used to monitor the progression of disease states in cases of autoimmunity (39, 40). TCR repertoires from *Lig4^W447C/W447C^* mice displayed increased clonality and decreased diversity compared with those from *Lig4^+/+^* mice (Supplemental Figure 12). Among expanded T cell clones, activated Th1 cells were dominant, whereas *Il4*- or *Il17a*-expressing T cells were not observed. Further clarification of these T cell clones, some of which may have pathogenic potential, should contribute to effective treatment for the inflammation under adaptive immunodeficiency.

In *Rag2^R229Q/R229Q^* and *Lig4^R278H/R278H^* mice, splenic T cells showed oligoclonal expression of TCR Vβ gene segments, but their usage of Vα was unclear (19, 35). In *Lig4^W447C/W447C^* mice, scRNA-seq analysis revealed preferential usage of proximal Vα and Jα gene segments. This skewed usage was not observed in Vβ and Jβ segments. This is consistent with the usage pattern of TCRα gene segments not only from Omenn or Omenn-like syndrome patients (41, 42), but also from the mutant mice lacking XLF, which stimulates the DNA-joining activity of the XRCC4-LIGIV complex (43). It would be interesting to determine whether T cells with these skewed TCRαs are involved in intestinal inflammation.

In this study, mutant mice carrying a hypomorphic *Lig4* mutation derived from a LIG4 syndrome patient showed T cell–dependent intestinal inflammation under severe adaptive immunodeficiency. The inflammation depends on activation and/or selection of Th cells, especially Th1 cells. The phenotype is based on the monogenic mutation and can account for the inflammation that accompanies LIG4 syndrome, but the underlying pathological mechanisms should have some aspects in common with polygenic inflammatory bowel diseases, which are still intractable. Our mutant mice should be a useful model to analyze and treat not only LIG4 syndrome itself, but also some common inflammatory conditions based on T cell activation and/or selection under adaptive immune deficiency.

## Methods

### Sex as a biological variable.

Both male and female mice were used in this study, because *Lig4^W447C/W447C^* mice invariably develop growth retardation, intestinal inflammation, and immunodeficiency regardless of sex.

### Mice.

*Lig4^W447C/W447C^*, *Ifng^−/−^*, *Lig4^W447C/W447C^ Rag2^−/−^*, and *Lig4^W447C/W447C^ Ifng^−/−^* mice were generated as described in Supplemental Methods. *Rag2^−/−^* mice were provided by Frederick W. Alt (Harvard Medical School, Boston, Massachusetts, USA) (44). C57BL/6N mice were purchased from CLEA Japan. All mice were housed in specific pathogen–free conditions with 12-hour light/12-hour dark cycles at an ambient temperature of 20°C–24°C and a humidity range of 40%–60%. Experimental and control animals were cohoused and used at 8–16 weeks old unless otherwise noted. Both males and females were used in the study. Mice were euthanized by cervical dislocation before tissue dissection.

### γH2AX foci assay.

MEFs were plated at a density of 1 × 10^5^ on coverslips and incubated overnight. Then, the cells were irradiated with 1 Gy of x-rays by an x-ray machine (OM-B205, Ohmic) operated at 70 kVp and 5 mA using a 0.5 mm Al filter at room temperature. The dose rate was 0.64 Gy/min. After x-irradiation, the cells were incubated at 37°C for 10 minutes and 0.5, 1, 3, 6, and 24 hours, then fixed with 4% paraformaldehyde phosphate buffer (Nacalai Tesque) for 15 minutes and permeabilized with 0.5% Triton X-100 (FUJIFILM Wako) in PBS (Gibco, Thermo Fisher Scientific) on ice for 5 minutes. The fixed cells were soaked with blocking buffer containing 5% Blocking One (Nacalai Tesque) and 0.1% Tween 20 (Sigma-Aldrich) in PBS (-) for 1 hour. The blocked cells were incubated with an anti–phospho–histone H2AX (Ser139) mouse monoclonal antibody conjugated with Alexa Fluor 488 (BioLegend) for 1 hour at room temperature. After washing with 0.1% Tween 20, the cells were counterstained with 4′,6-diamidino-2-phenylindole (DAPI; Sigma-Aldrich), and coverslips were placed on glass slides followed by sealing with clear nail polish. For accurate and reproducible counting of the number of γH2AX foci, fluorescence images were acquired using a fluorescence microscope (BX-51, Olympus) equipped with a digital camera (DP71, Olympus), and analyzed with a newly developed ImageJ-based computer program, as previously described (45).

### Western blot analysis.

In brief, MEFs from *Lig4*-mutated mice were lysed with radioimmunoprecipitation assay lysis buffer (Nacalai Tesque) and cOmplete Mini Protease Inhibitor Cocktail (Roche Diagnostics). The protein extracts were mixed with Laemmli sample buffer containing 2-mercaptoethanol (Wako) and heated in a heat block at 100°C for 5 minutes. The samples were separated using sodium dodecylsulfate–polyacrylamide gel electrophoresis and transferred onto polyvinylidene fluoride membranes (GE Healthcare). The membranes were washed and blocked with the DIG Wash and Block Buffer Set (Roche Diagnostics) according to the manufacturer’s instructions. The membranes were then incubated overnight at 4°C with primary antibodies. The following primary antibodies were used: rabbit anti-LIG4 (catalog ab26039, Abcam), mouse anti-XRCC4 (catalog MA5-24383, Thermo Fisher Scientific), rabbit anti-XLF (catalog ab33499, Abcam), and mouse anti–β-actin antibodies (catalog 3700, Cell Signaling Technology). The following day, after 3 washes, the membranes were incubated with secondary antibodies at room temperature for 1 hour. The following secondary antibodies were used: horseradish peroxidase–conjugated goat anti-rabbit IgG polyclonal antibody (1:1,000 dilution; catalog 170-6515, Bio-Rad) and horseradish peroxidase–conjugated rabbit anti-mouse IgG antibody (1:1,000 dilution; catalog 1080-05, SouthernBiotech). The protein bands were visualized with SuperSignal West Dura Substrate (Thermo Fisher Scientific) and detected using a LuminoGraph I Chemiluminescent Imaging System (ATTO). We measured the band intensities using ImageJ software version 1.51k (NIH). The β-actin level was used as an internal control to ensure equal amounts of loading proteins.

### LIGIV protein structure predictive analysis.

Atomic models of wild-type and mutated LIGIV proteins were predicted with AlphaFold 3 web server with default settings (46). The amino acid sequence of mouse LIGIV was used as the input. Five models were generated, and the relaxed model with the highest confidence score was selected for analysis. The molecular graphics were made using PyMOL (http://www.pymol.org/).

### Cell-free in vitro DNA end-joining assay.

NHEJ activity was assessed using a cell-free in vitro DNA end-joining assay with linearized plasmid substrates, as previously described with minor modifications (47, 48). Immortalized wild-type and *Lig4* mutant cells were lysed using M-PER Mammalian Protein Extraction Reagent (Thermo Fisher Scientific), and protein concentrations were quantified before storage at –80°C. Cell extract protein was incubated with restriction enzyme–linearized plasmid DNA in T4 DNA ligase reaction buffer (New England Biolabs), which provides optimal ionic conditions (including ATP and Mg^2+^) to support LIG4-dependent end joining. In brief, the pUCF-based plasmid (FASMAC) containing a BamH1 recognition site was linearized by digestion with BamHI to generate cohesive ends. After incubation of 70 ng of linearized plasmid with 20 ng of cell extract in 20 µL of reaction buffer, quantitative PCR was performed using primers designed to selectively amplify re-ligated circular plasmid DNA. Absolute copy numbers were calculated based on a standard curve generated from known amounts of circular plasmid, and end-joining efficiency was expressed as the percentage of input linear plasmid converted to circular DNA.

### Histological examination.

The thymus, spleen, small intestine, and colon were formalin fixed, paraffin embedded, processed, and sectioned into 5-μm-thick sections. In addition, the BM was pre-decalcified. The sections were stained with hematoxylin and eosin (H&E). Colitis and enteritis were histomorphologically assessed. Colitis was assessed using the MCHI rating scale, as previously described by Koelink et al. (49). The total MCHI score ranged from 0 (no disease) to 22 (severe disease) and was calculated using 4 categories (goblet cell loss, crypt density, hyperplasia, and submucosal invasion) as follows: MCHI = 1 × goblet cell loss (0 to 3) + 2 × crypt density (0 to 2) + 2 × hyperplasia (0 to 3) + 3 × submucosal invasion (0 to 3). Enteritis was assessed using different criteria, as described in Supplemental Methods. Furthermore, we performed immunohistochemical analysis as previously described (50). Briefly, the thymus, spleen, small intestine, and colon were formalin fixed, paraffin embedded, and processed to obtain 5-μm-thick sections. In addition, we performed antigen retrieval using a microwave and a citrate buffer (pH 6.0; Sigma-Aldrich). Then, tissue samples were incubated at room temperature for 60 minutes with primary antibodies. The following primary antibodies were used: anti–mouse CD3 antibody (CD3-12, Abcam), rabbit anti–mouse CD4 antibody (EPR19514, Abcam), anti–mouse F4/80 antibody (D2S9R, Cell Signaling Technology), and anti–mouse B220 antibody (RA3-6B2, BD). Next, 3,3′-diaminobenzidine (DAB; Sigma-Aldrich) staining was performed using the 2-step EnVision+ System–HRP methodology (Dako, Tokyo, Japan). For visualization, light microscopy was performed using an Olympus BX43 microscope (Olympus). Virtual slides were generated using the NanoZoomer S60 C13210 (Hamamatsu Photonics), and digitized whole-slide images were acquired. Images for analysis were subsequently extracted using NDP.view2Plus software (Hamamatsu Photonics).

Neutrophils, eosinophils, and multinucleated giant cells were identified morphologically in H&E-stained sections, whereas macrophages were identified by immunohistochemistry using an anti-F4/80 antibody. The number of each cell type per high-power field (×400) was quantified in hotspot areas of the intestinal LP. Mast cells were quantified using toluidine blue (Toluidine Blue O, Sigma-Aldrich) staining. 

### Cell preparations.

Thymocyte, splenocyte, and MLN suspensions were prepared by grinding of the appropriate organs through mesh filters. In addition, BM cells were extracted from the femur and tibia and passed through mesh filters. Furthermore, intestinal immune cells were prepared from the small intestine. In brief, fat and Peyer’s patches were dissected away before the small intestine was opened longitudinally and stirred in RPMI 1640 medium (Nacalai Tesque) containing 2% fetal bovine serum (JR Scientific Inc.) and 2 mM EDTA (Nacalai Tesque) for 20 minutes at 37°C. Intraepithelial lymphocytes were isolated from the supernatant using a 40%–75% Percoll density gradient (GE Healthcare) by collection of cells that layered between the 40% and 75% fractions. After supernatant collection, the intestinal tissue was stirred for 20 minutes at 37°C in RPMI 1640 medium (Nacalai Tesque) containing 2% fetal bovine serum before mincing and further stirring in 400 U/mL of collagenase D (Roche Diagnostics) and 10 μg/mL of DNase I (Sigma-Aldrich) for 20 minutes at 37°C. Floating cells were collected, and the collagenase digestion was repeated twice more. The pooled cell suspensions were then centrifuged on a 40%–75% Percoll density gradient, and cells that layered between the 40%–75% fractions were collected as LP cells.

### Flow cytometric analysis.

A single-cell suspension was prepared from the thymus, spleen, BM, MLNs, and LP cells and analyzed using flow cytometry according to standard protocols. The LIVE/DEAD Fixable Aqua Dead Cell Stain Kit (Invitrogen) was used to exclude dead cells. The anti-CD16/32 antibody (2.4G2, Tonbo Biosciences) was used to block Fc receptors, and the cells were then stained with fluorochrome-conjugated antibodies (Supplemental Table 6). For S1PR1 staining, cells were first incubated with an unconjugated anti-S1PR1 antibody or the corresponding isotype control, followed by Alexa Fluor 647–conjugated chicken anti-rabbit IgG antibody (Supplemental Table 6). After incubation with 2% normal rat serum (Jackson ImmunoResearch) in PBS, cells were subsequently stained with fluorochrome-conjugated antibodies against other markers. For intracellular staining of IFN-γ, IL-17A, and IL-4, splenocytes, MLN cells, and LP cells were stimulated with PMA (50 ng/mL; Nacalai Tesque), ionomycin (500 ng/mL; Nacalai Tesque), and GolgiPlug (BD Biosciences) for 5 hours. Cells were stained with monoclonal antibodies against TCRβ, CD4, and CD8α, then fixed with fixation/permeabilization solution (Cytofix/Cytoperm Kit, BD Biosciences) and stained with anti–IFN-γ, anti–IL-17A, or anti–IL-4 monoclonal antibodies or the corresponding isotype control (Supplemental Table 6). Foxp3 Staining Buffer Set (eBioscience) was used to stain Foxp3, T-bet, GATA3, and RORγt. Stained cells were analyzed using a FACSVerse flow cytometer (BD Biosciences), and data were analyzed using FlowJo software version 10.8.0 (BD Biosciences). Representative flow cytometry gating strategies, including fluorescence minus one (FMO) controls used to define gating boundaries, are provided in Supplemental Figure 13.

### Measurement of serum Ig levels in mice.

Serum Ig levels of *Lig4^W447C/W447C^* mice and wild-type C57BL/6N littermates were measured using an ELISA, as previously described (51). In brief, serum samples were incubated in an ELISA plate (Thermo Fisher Scientific) coated with anti-mouse IgM, IgG1, IgA, or IgE antibodies and detected with biotinylated antibodies against each class and streptavidin-conjugated alkaline phosphatase (SouthernBiotech). The plate was then developed using an alkaline phosphatase buffer (NaHCO_3_ [50 mM], MgCl_2_ [10 mM], pH 9.8) containing a phosphatase substrate tablet (Sigma-Aldrich). Purified mouse IgM, IgG1, IgA, and IgE were used as standards. Details of all antibodies and standards are listed in Supplemental Table 7.

### Mouse BM transplantation.

BM cells were isolated from the femora and tibiae of *Lig4^W447C/W447C^* mice and wild-type C57BL/6N littermates (9–22 weeks of age). BM cells (0.5 × 10^7^ to 1.0 × 10^7^) were injected via the tail vein into *Rag2^−/−^* recipient mice (10–30 weeks of age) that had received a lethal total-body γ-irradiation dose of 10 Gy. Mice were monitored daily for survival and weighed twice a week. Euthanasia was performed 7 weeks after transplantation for analysis.

### Bulk RNA sequencing analysis.

For RNA-seq analysis, total RNA was extracted using the RNeasy Micro Kit (QIAGEN) or Sepasol-RNA I Super G (Nacalai Tesque), as previously described (52, 53). The samples were collected from the colon, MLNs, and spleen of *Lig4^+/+^* and *Lig4^W447C/W447C^* mice and from the colon of *Lig4^W447C/W447C^ Ifng^−/−^* mice. The RNA quality was assessed using an Agilent 4200 TapeStation (Agilent Technologies), and the RNA concentration was measured using a Qubit Fluorometer (Thermo Fisher Scientific). A total of 4,000 ng RNA in colon, 228–1,552 ng RNA in MLNs, and 921.6–3,000 ng RNA in spleen was used, and libraries for sequencing were constructed using TruSeq Stranded mRNA (Illumina Inc.) according to the manufacturer’s protocol. The concentration of libraries was estimated using the KAPA Library Quantification Kit (Roche Diagnostics). The range of average library size was 324–359 bp. The libraries were sequenced by high-throughput sequencing using a NextSeq 500/550 High Output Kit v2.5 (Illumina; 75 cycles paired-end, 40/40 cycles). The average number of sequence reads per sample was 22,855,222.1. The bulk RNA-seq results were analyzed using the CLC Genomics Workbench version 12.0.2 (Filgen Inc.), and the abundance of gene expression was expressed using the reads per kilobase per million (RPKM) values. In addition, gene expression analysis, including GSEA, was conducted using GSEA v4.2.3 software and the Molecular Signatures Database v7.5.1 (54, 55). Moreover, the web tools ClustVis and BioJupies were used to draw a heatmap and a volcano plot, respectively (56, 57).

### Single-cell RNA-sequencing and TCR repertoire analysis.

Single-cell transcriptome and TCR repertoire analysis was performed using Chromium Single Cell 5′ Reagent Kits v2 and Chromium Single Cell Mouse TCR Amplification Kit with Chromium Controller (10x Genomics) according to the manufacturer’s instructions as previously described (58). Libraries were sequenced on NovaSeq X Plus (Illumina) in paired-end mode (read 1, 151 bp; read 2, 151 bp). The raw reads were processed by Cell Ranger v7.1.0 (10x Genomics). Gene expression–based clustering was performed using the Seurat R package (v3.1). Cells with a mitochondrial content greater than 10% and cells with fewer than 200 or more than 4,000 genes detected were considered outliers (dying cells and empty droplets and doublets, respectively) and filtered out. The Seurat SCTransform function was used for normalization, and data were integrated without batch-effect correction as all samples were processed simultaneously. Hashtag oligonucleotide demultiplexing was performed on centered log ratio–normalized hashtag unique molecular identifier counts, and clonotypes were matched to the gene expression data through their droplet barcodes, using Python scripts. The cluster analysis based on t-distributed stochastic neighbor embedding (t-SNE) plot and differential expression analysis were performed by Loupe Browser v8.0.0 (10x Genomics). The TCR repertoire analysis was performed by Loupe V(D)J Browser v5.2.0 (10x Genomics).

### Statistics.

Statistical analysis was performed using GraphPad Prism 9 (GraphPad Software). The data were compared using an unpaired, 2-tailed Student’s *t* test, unless otherwise specified, and are presented as the mean and SEM. *P* values less than 0.05 were considered statistically significant.

### Study approval.

All animal experiments were approved by the Animal Research Committee of Wakayama Medical University (approval 855 and 1140) and conducted in accordance with the Guidelines for the Care and Use of Laboratory Animals at Wakayama Medical University, as well as the relevant national guidelines and regulations.

### Data availability.

Values for all data points associated with the figures and supplemental figures are provided in the Supporting Data Values file. The raw FASTQ data of RNA-seq analysis were deposited in DNA Data Bank of Japan under BioProject number PRJDB19195 and run number DRR615557–DRR615579. The single-cell RNA-seq data generated in this study were deposited in the NCBI’s Gene Expression Omnibus (GEO) database under accession number GSE294169. In addition, processed scRNA-seq expression matrices (Supplemental Tables 8–11) and TCR clonotype tables (Supplemental Tables 12–15) for each mouse are provided as supplemental files. Requests for materials and for additional information should be addressed to Yusuke Yamashita, Tsuneyasu Kaisho, or Shinobu Tamura.

## Author contributions

YY, S Tamura, and T Kaisho designed the research. YY, HK, T Kato, IS, TO, MT, S Tabata, KN, KT, KS, YU, AS, SK, HM, KO, and YF performed biochemical and histochemical experiments. TM performed in silico analyses on the LIGIV protein structures. YY, HH, T Kaisho, and S Tamura generated and analyzed murine models. YFO and KI contributed to the generation of *Lig4^W447C/W447C^* mouse and *Ifng^−/−^* mouse models, respectively, using the CRISPR/Cas9 system. YY, HK, TO, MT, S Tabata, YFO, and KI contributed to the maintenance of mice. YY, HK, SI, DO, and SH performed genomic analyses. YY, NK, TS, T Kaisho, and S Tamura wrote and edited the manuscript. YY and HK contributed equally to this work. The order of co–first authors was determined based on the relative contributions to experimental design, data acquisition, and manuscript preparation.

## Conflict of interest

The authors have declared that no conflict of interest exists.

## Funding support

- Grants-in-Aid for Transformative Research Areas (JP22H05182 and JP22H05187 to T Kaisho and JP22H05187 to IS), for Scientific Research (B) (JP26293106, JP17H04088, JP20H03505, and JP24K02298 to T Kaisho), for Scientific Research (C) (JP19K07628 and JP22K07006 to IS, JP21K12243 to SK, JP19K08821 to TS, and JP20K08718 and JP23K07865 to S Tamura), for Scientific Research on Innovative Areas (JP17H05799 and JP19H04813 to T Kaisho), for Early-Career Scientists (JP20K17405 and JP22K16308 to YY and JP25K19582 to HK), for Research Activity Start-Up (JP23K19487 to T Kato), for JSPS Fellows (JP21J22615 to T Kato), and for Exploratory Research (JP17K19568, JP21K19384, and JP23K18222 to T Kaisho) from the Japan Society for the Promotion of Science (JSPS).
- Takeda Science Foundation (to YY, IS, HH, and T Kaisho).
- GSK Japan Research Grant 2021 (to IS).
- Kowa Life Science Foundation (to IS).
- Wakayama Medical University Special Grant-in-Aid for Research Projects (K23TS03 to S Tamura).

## Supplementary Material

Supplemental data

Unedited blot and gel images

Supplemental table 1

Supplemental table 10

Supplemental table 11

Supplemental table 12

Supplemental table 13

Supplemental table 14

Supplemental table 15

Supplemental table 2

Supplemental table 3

Supplemental table 4

Supplemental table 5

Supplemental table 6

Supplemental table 7

Supplemental table 8

Supplemental table 9

Supporting data values

## Figures and Tables

**Figure 1:**
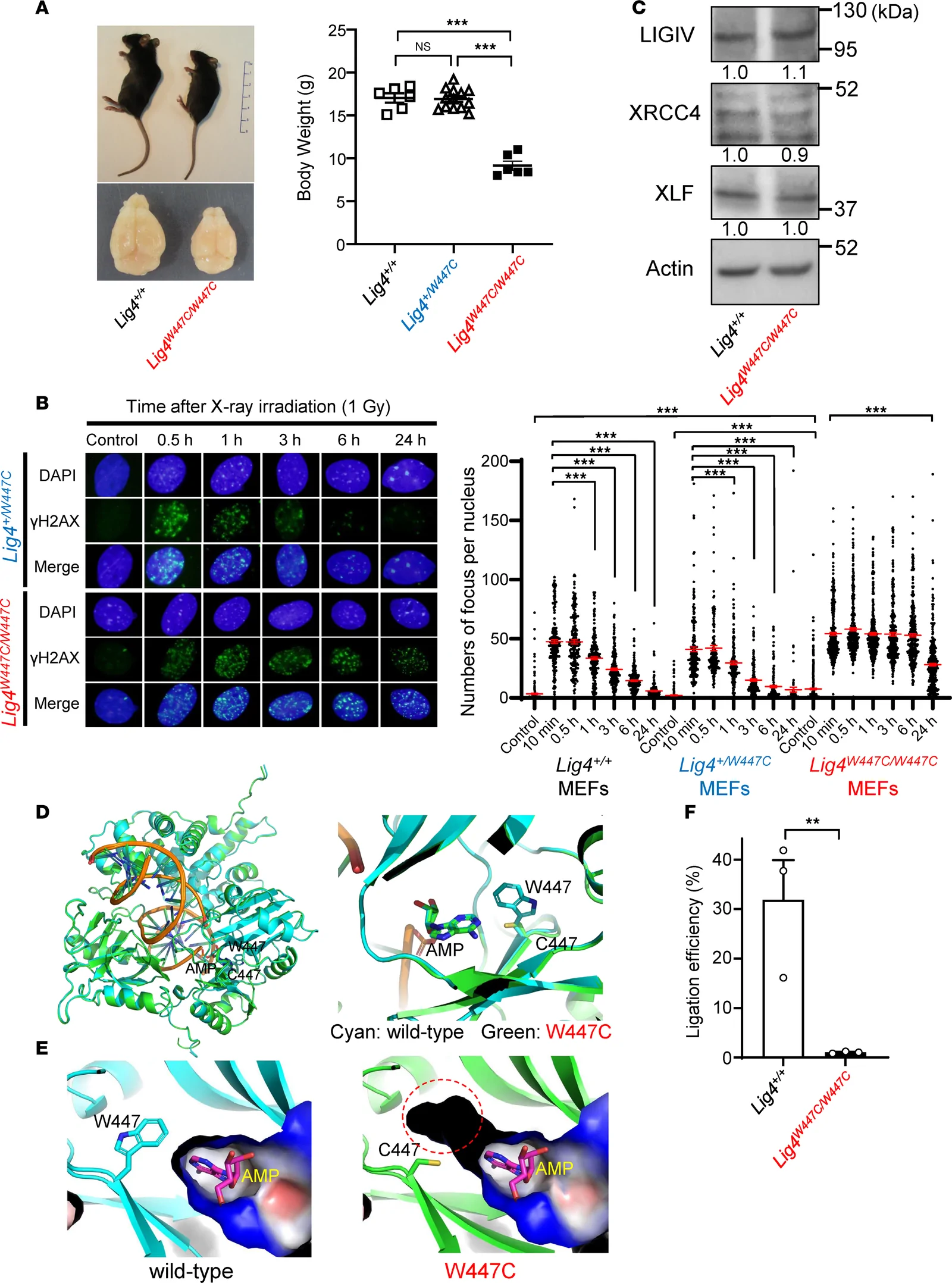
*Lig4^W447C/W447C^* mice displayed typical LIG4 syndrome features. (**A**) Appearance and brain of 6-week-old *Lig4^+/+^* and *Lig4^W447C/W447C^* littermates. The graph depicts the weight of 6-week-old female mice (*Lig4^+/+^*, *n* = 6; *Lig4^+/W447C^*, *n* = 13; *Lig4^W447C/W447C^*, *n* = 6). *P* values were calculated using 1-way ANOVA followed by Tukey’s post hoc multiple-comparison test. (**B**) Immunofluorescence analysis of γH2AX foci in MEFs after 1 Gy irradiation. Left: Representative images of γH2AX foci in the nuclei of MEFs at the indicated time points. Right: Quantification of γH2AX foci in plateau-phase MEFs after 1 Gy irradiation. Sample sizes ranged from 119 to 391 cells per group. Within each genotype, 1-way ANOVA was conducted, followed by Tukey’s multiple-comparison test. Mann-Whitney *U* test was used to compare baseline measurements among different genotypes. Original magnification: ×100. (**C**) Western blots of indicated protein expressions on MEFs. Numbers below the representative bands indicate the ratio of signal intensity relative to *Lig4^+/+^* after normalization to β-actin. (**D**) Left: Mouse LIGIV protein structures in wild-type and W447C mutant mice. Right: The overall LIGIV protein structure, with DNA-binding and enzymatic domains (residues 1–620), is depicted as a ribbon model. W447 and C447 binding to AMP is shown in stick representation (cyan and green, respectively). (**E**) Structural comparison between W447 (left, cyan) and C447 (right, green). AlphaFold 3–predicted side chains related to AMP binding are substantially different between them. Consequently, W447C leads to enlargement of the AMP-binding pocket (dashed red circle in right panel), suggesting destabilization of the AMP-binding site during DNA repair processes (black region). (**F**) Ligation efficiency in an in vitro plasmid-based DNA end-joining assay. Ligation efficiency was quantified by quantitative PCR detection of re-ligated circular plasmid DNA in MEF extracts. Data represent results of 3 independent experiments. In **A**, **B**, and **F**, data are presented as mean ± SEM. **P < 0.01, ***P < 0.001.

**Figure 2:**
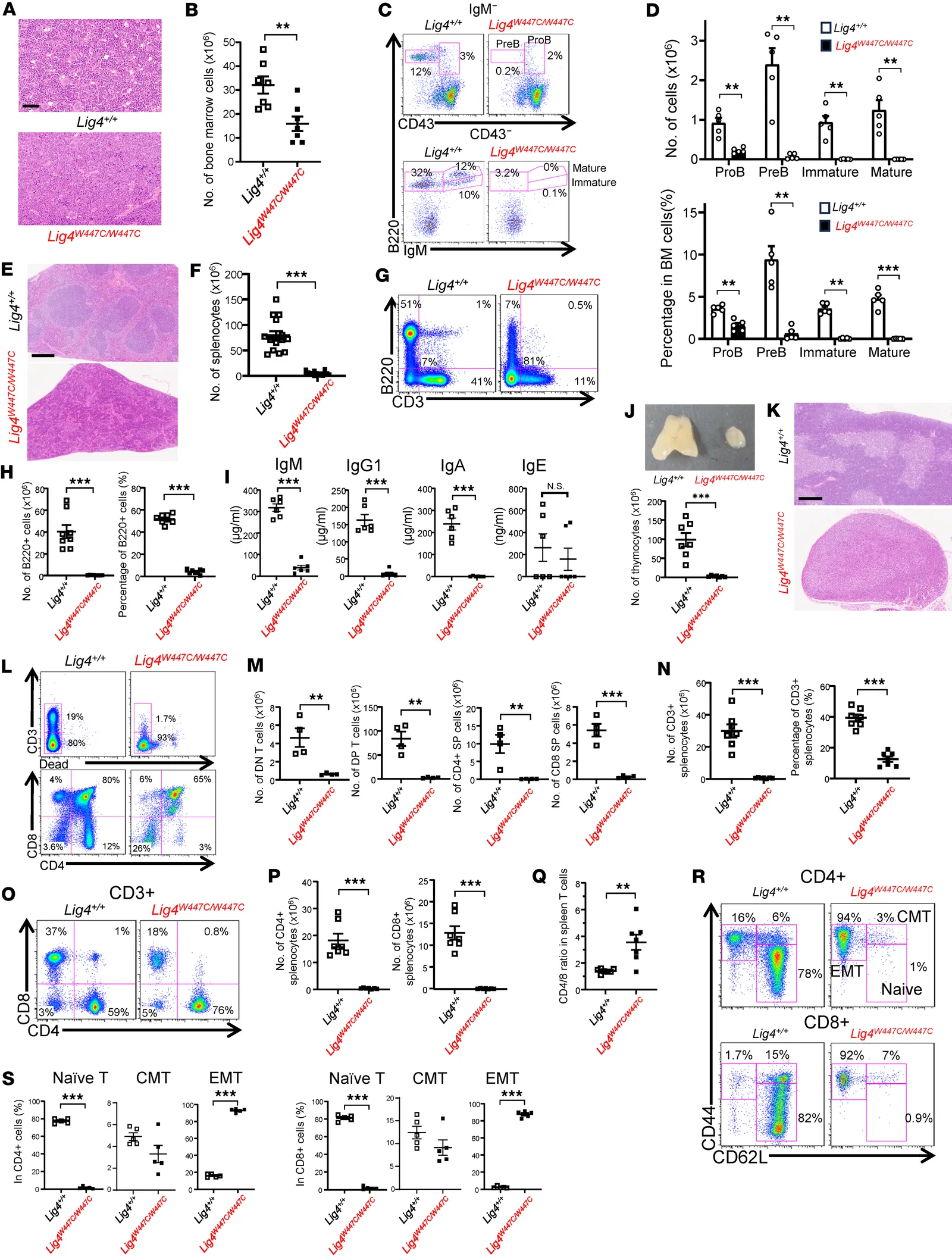
B and T cell differentiation was impaired in *Lig4^W447C/W447C^* mice. (**A**) H&E staining revealed hypocellular marrow in BM of *Lig4^W447C/W447C^* mice compared with that of *Lig4^+/+^* mice. Scale bar: 60 μm. (**B**) Number of BM cells (*Lig4^+/+^*, *n* = 7; *Lig4^W447C/W447C^*, *n* = 7; 7–23 weeks). (**C**) FACS analysis of BM cells labeled with B220, IgM, and CD43 antibodies. (**D**) Number and percentage of B cells in BM (*Lig4^+/+^*, *n* = 5; *Lig4^W447C/W447C^*, *n* = 5; 8–30 weeks). (**E**) H&E staining of spleen. Scale bar: 300 μm. (**F**) Splenocyte numbers (*Lig4^+/+^*, *n* = 15; *Lig4^W447C/W447C^*, *n* = 15; 7–20 weeks). (**G**) FACS analysis of splenocytes labeled with CD3 and B220 antibodies. (**H**) Absolute number and percentage of B220^+^ splenocytes analyzed by FACS (*Lig4^+/+^*, *n* = 8; *Lig4^W447C/W447C^*, *n* = 8; 7–20 weeks). (**I**) Serum Ig levels (*Lig4^+/+^*, *n* = 6; *Lig4^W447C/W447C^*, *n* = 6; 9–12 weeks). (**J**) Thymic hypoplasia in a 6-week-old *Lig4^W447C/W447C^* mouse. The graph depicts the number of thymocytes (*Lig4^+/+^*, *n* = 7; *Lig4^W447C/W447C^*, *n* = 7; 8–23 weeks). (**K**) H&E staining of thymus. Scale bar: 300 μm. (**L**) FACS analysis of thymocytes labeled with CD3, CD4, and CD8 antibodies. (**M**) T cell subset numbers in thymocytes, including DN, DP, and SP (*Lig4^+/+^*, *n* = 4; *Lig4^W447C/W447C^*, *n* = 4; 7–16 weeks). (**N**) Number and percentage of CD3^+^ splenocytes (*Lig4^+/+^*, *n* = 7; *Lig4^W447C/W447C^*, *n* = 7; 8–11 weeks). (**O**) FACS analysis of splenocytes labeled with CD3, CD4, and CD8 antibodies. (**P**) Absolute numbers of CD4^+^ and CD8^+^ splenocytes (*Lig4^+/+^*, *n* = 7; *Lig4^W447C/W447C^*, *n* = 7; 8–32 weeks). (**Q**) CD4/CD8 ratio of CD3^+^ spleen T cells (*Lig4^+/+^*, *n* = 7; *Lig4^W447C/W447C^*, *n* = 7; 8–32 weeks). (**R**) FACS analysis of CD44 and CD62L in splenic CD4^+^ and CD8^+^ T cells. (**S**) Percentages of naive, central memory (CMT), and effector memory (EMT) T cell subsets (*Lig4^+/+^*, *n* = 5; *Lig4^W447C/W447C^*, *n* = 5; 9–10 weeks). Data are presented as mean ± SEM. **P < 0.01, ***P < 0.001.

**Figure 3:**
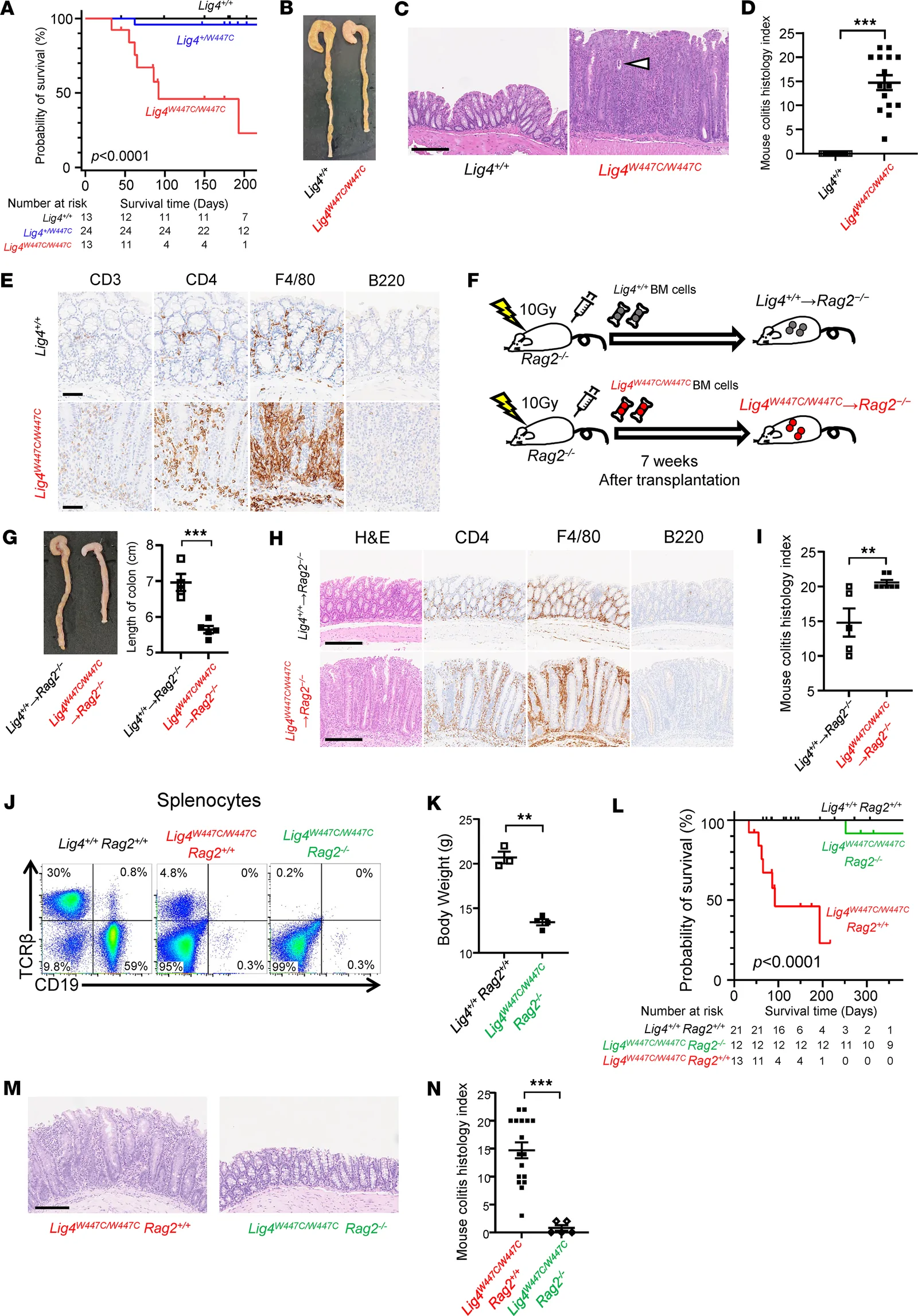
Hematopoietic cells caused severe intestinal inflammation in *Lig4^W447C/W447C^* mice. (**A**) Kaplan-Meier survival curves of *Lig4^+/+^* (*n* = 13), *Lig4^+/W447C^* (*n* = 24), and *Lig4^W447C/W447C^* (*n* = 13) mice. Statistical analysis was performed using the log-rank test. (**B**) Macroscopic features of the *Lig4^W447C/W447C^* mouse colon revealed an edematous, thickened, and shortened colon. (**C**) Representative H&E staining of colons. White arrowhead indicates a crypt abscess. Scale bar: 200 μm. (**D**) Mouse colitis histology index (MCHI) (*Lig4^+/+^*, *n* = 15; *Lig4^W447C/W447C^*, *n* = 15; 7–13 weeks). (**E**) Immunohistochemistry of colons with CD3, CD4, F4/80, and B220 antibodies. Scale bars: 60 μm. (**F**) Scheme of BM transplantation experiment. BM cells from *Lig4^+/+^* or *Lig4^W447C/W447C^* mice were transplanted into irradiated *Rag2^−/−^* mice. (**G**) A shortened and edematous colon from a *Lig4^+/+^*→*Rag2^−/−^* mouse and a *Lig4^W447C/W447C^*→*Rag2^−/−^* mouse. The graph depicts the length of colons. (*Lig4^+/+^*→*Rag2^−/−^*, *n* = 4; *Lig4^W447C/W447C^*→*Rag2^−/−^*, *n* = 5). (**H**) H&E staining and immunohistochemistry with CD4, F4/80, and B220 antibodies of colons from a *Lig4^+/+^*→*Rag2^−/−^* mouse and a *Lig4^W447C/W447C^*→*Rag2^−/−^* mouse. Scale bars: 100 μm. (**I**) MCHI of *Lig4^+/+^*→*Rag2^−/−^* and *Lig4^W447C/W447C^*→*Rag2^−/−^* mice (*Lig4^+/+^*→*Rag2^−/−^*, *n* = 5; *Lig4^W447C/W447C^*→*Rag2^−/−^*, *n* = 7). (**J**) FACS analysis of CD19 and TCRβ expression in splenocytes. (**K**) Weight of female mice (*Lig4^+/+^ Rag2^+/+^*, *n* = 3; *Lig4^W447C/W447C^* *Rag2^−/−^*, *n* = 4; 14–16 weeks). (**L**) Kaplan-Meier survival curves of *Lig4^+/+^ Rag2^+/+^* (*n* = 21), *Lig4^W447C/W447C^ Rag2^+/+^* (*n* = 13), and *Lig4^W447C/W447C^ Rag2**^−/−^*** (*n* = 12) mice. Statistical analysis was performed using the log-rank test. (**M**) Representative H&E staining of colons. Scale bar: 200 μm. (**N**) MCHI (*Lig4^W447C/W447C^ Rag2^+/+^*, *n* = 17; *Lig4^W447C/W447C^ Rag2^−/−^*, *n* = 5; 7–18 weeks). Data are presented as mean ± SEM. **P < 0.01, ***P < 0.001.

**Figure 4:**
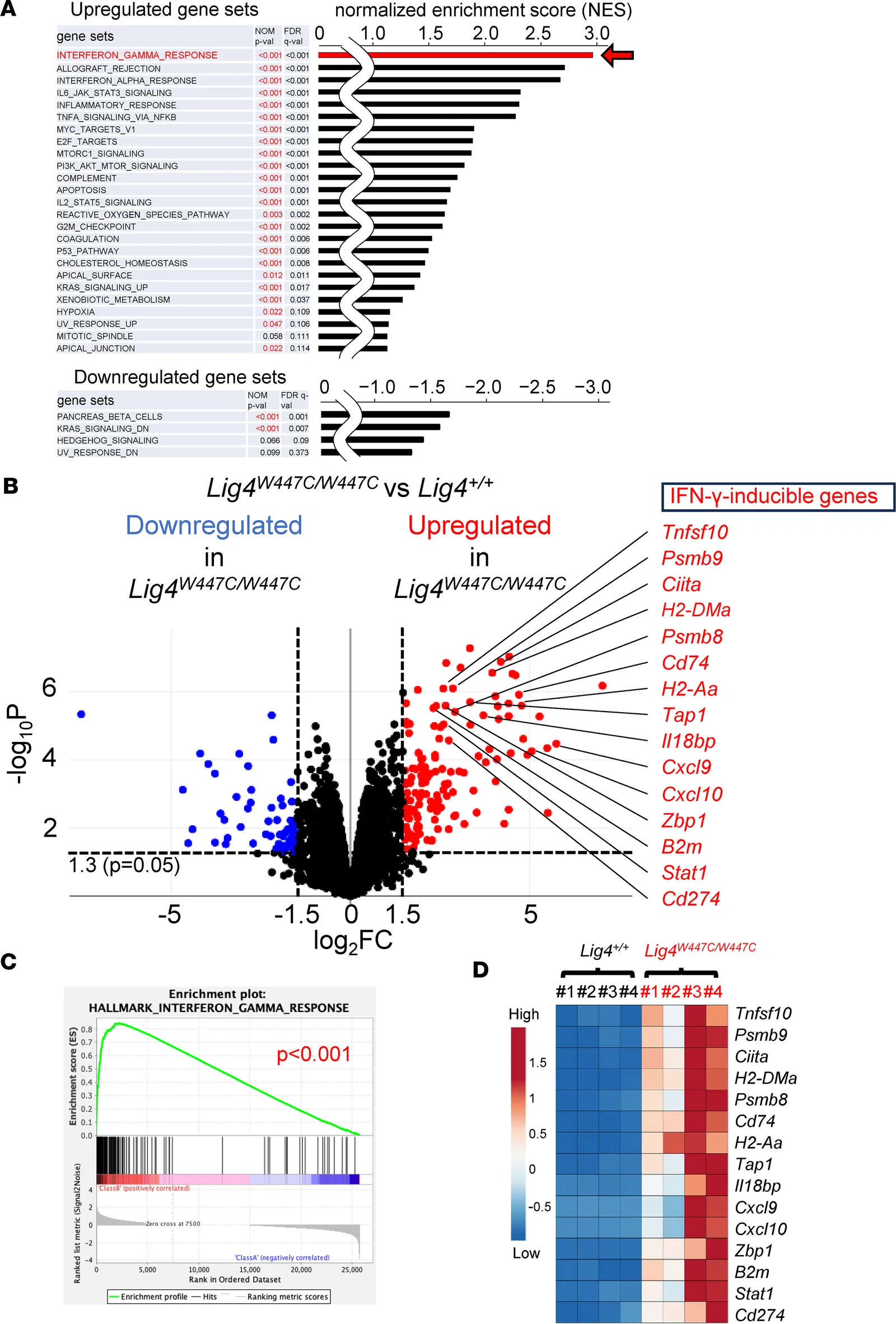
Bulk RNA-seq analysis of the colon of *Lig4^W447C/W447C^* mice. (**A**) Gene set enrichment analysis (GSEA) revealed the top 25 upregulated (top) and top 4 downregulated (bottom) gene sets in the colons of *Lig4^W447C/W447C^* mice compared with *Lig4^+/+^* mice, based on normalized enrichment scores. Gene sets associated with “interferon-gamma response,” indicated by the red arrow, represent the focus of this study. NOM p-val, nominal *P* value; FDR q-val, false discovery rate *q* value. (**B**) Relative expression levels in the colons of eight 10-week-old mice (*Lig4^+/+^*, *n* = 4; *Lig4^W447C/W447C^*, *n* = 4) analyzed by RNA-seq analysis. Dashed lines indicate log_2_ fold-change thresholds of −1.5 and +1.5 (*x* axis) and *P* = 0.05 (*y* axis). (**C**) GSEA enrichment plot for “interferon-gamma response” comparing the colons of 4 *Lig4^W447C/W447C^* mice and 4 *Lig4^+/+^* mice. “p” indicates nominal *P* value. (**D**) Heatmap shows the changes in gene expression in the IFN-γ–inducible genes within the “interferon-gamma response” gene sets. Numbers with # represent individual mice.

**Figure 5:**
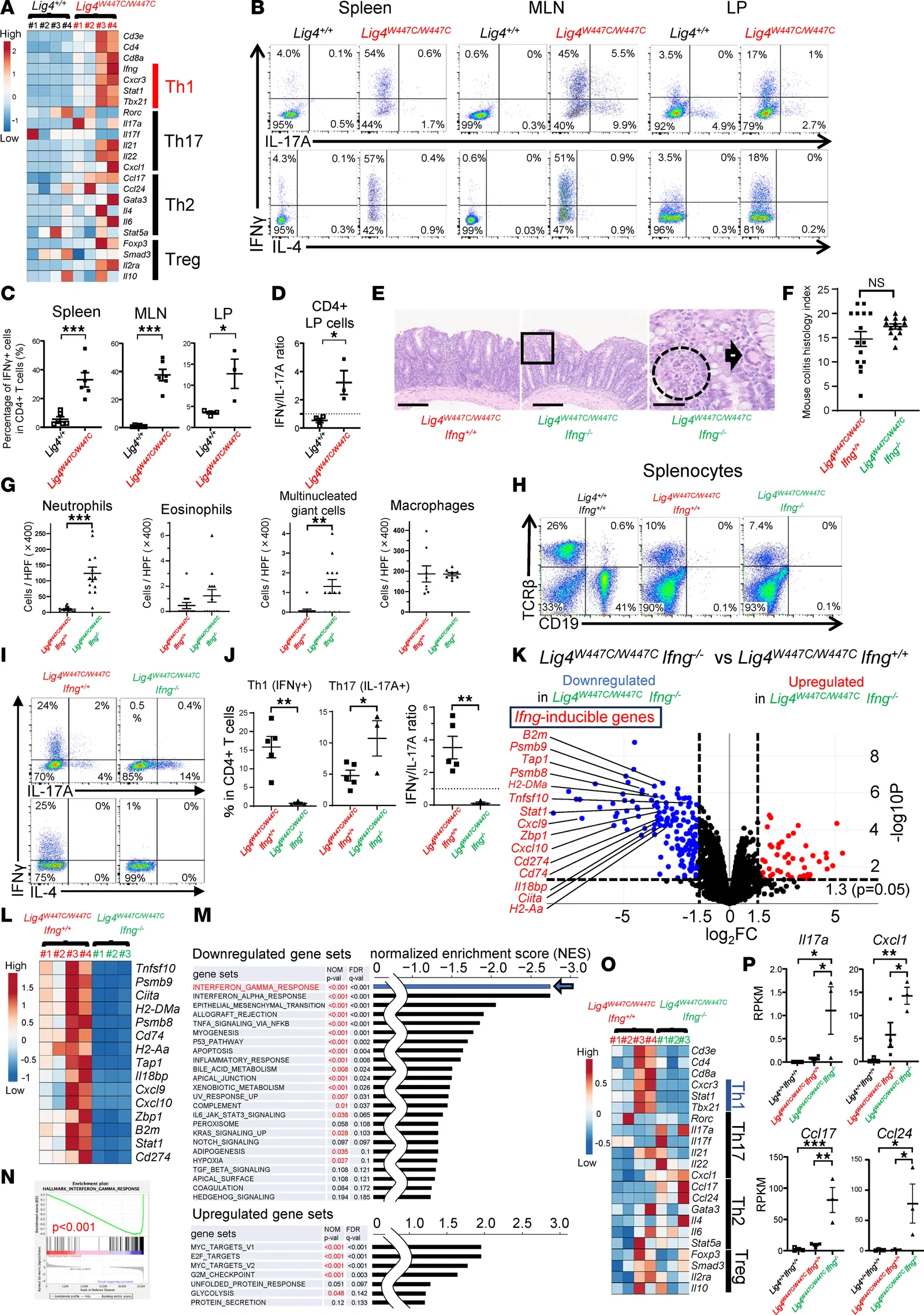
Analysis of *Lig4^W447C/W447C^ Ifng^−/−^* mice. (**A**) Heatmap of Th cell subset signature genes in colons of 10-week-old mice (*Lig4^+/+^*, *n* = 4; *Lig4^W447C/W447C^*, *n* = 4) analyzed by bulk RNA-seq. (**B**) Intracellular cytokine staining of CD4^+^ T cells in splenocytes, MLNs, and intestinal LP. (**C**) Percentages of IFN-γ–producing CD4^+^ T cells (*Lig4^+/+^*: spleen and MLNs, *n* = 6; LP, *n* = 4; *Lig4^W447C/W447C^*: spleen and MLNs, *n* = 6; LP, *n* = 3). (**D**) Ratio of IFN-γ/IL-17A–producing CD4^+^ T cells in LP. (**E**) H&E staining of colons. The square in the middle panel is magnified in the right panel. Dashed circle, infiltrated eosinophils; arrow, multinucleated giant cell. Scale bars: 200 μm (left and middle), 50 μm (right). (**F**) MCHI (*Lig4^W447C/W447C^ Ifng^+/+^*, *n* = 15; *Lig4^W447C/W447C^ Ifng^−/−^*, *n* = 13; 7–13 weeks). (**G**) Quantification of myeloid cells (*Lig4^W447C/W447C^ Ifng^+/+^*, *n* = 13; *Lig4^W447C/W447C^ Ifng^−/−^*, *n* = 13; F4/80^+^ cells, *n* = 8 each). (**H**) FACS analysis of CD19 and TCRβ expression in splenocytes. (**I**) Intracellular cytokine staining of CD4^+^ T cells in intestinal LP. (**J**) Percentages of IFN-γ^+^ and IL-17A^+^ CD4^+^ T cells in intestinal LP and their ratio (*Lig4^W447C/W447C^ Ifng^+/+^*, *n* = 5; *Lig4^W447C/W447C^ Ifng^−/−^*, *n* = 3). (**K**) Relative expression levels in colons of 10-week-old mice (*Lig4^W447C/W447C^ Ifng^+/+^*, *n* = 4; *Lig4^W447C/W447C^ Ifng^−/−^*, *n* = 3). Dashed lines indicate log_2_ fold-change thresholds of −1.5 and +1.5 (*x* axis) and *P* = 0.05 (*y* axis). (**L**) Heatmap of IFN-γ–inducible genes in the “interferon-gamma response” gene set in colons. (**M**) GSEA revealed the top 24 downregulated and top 7 upregulated gene sets in colons of *Lig4^W447C/W447C^ Ifng^−/−^* mice (*n* = 3) versus *Lig4^W447C/W447C^ Ifng^+/+^* mice (*n* = 4). Blue arrow, “interferon-gamma response” gene sets. NOM p-val, nominal *P* value; FDR q-val, false discovery rate *q* value. (**N**) GSEA enrichment plot for “interferon-gamma response” comparing colons of *Lig4^W447C/W447C^ Ifng^+/+^* mice (*n* = 4) and *Lig4^W447C/W447C^ Ifng^−/−^* mice (*n* = 3). “p” indicates nominal *P* value. (**O**) Heatmap of Th cell subset signature genes in colons. (**P**) Reads per kilobase per million (RPKM) levels of indicated genes in colons. Data are presented as mean ± SEM. *P < 0.05, **P < 0.01, ***P < 0.001.

**Figure 6:**
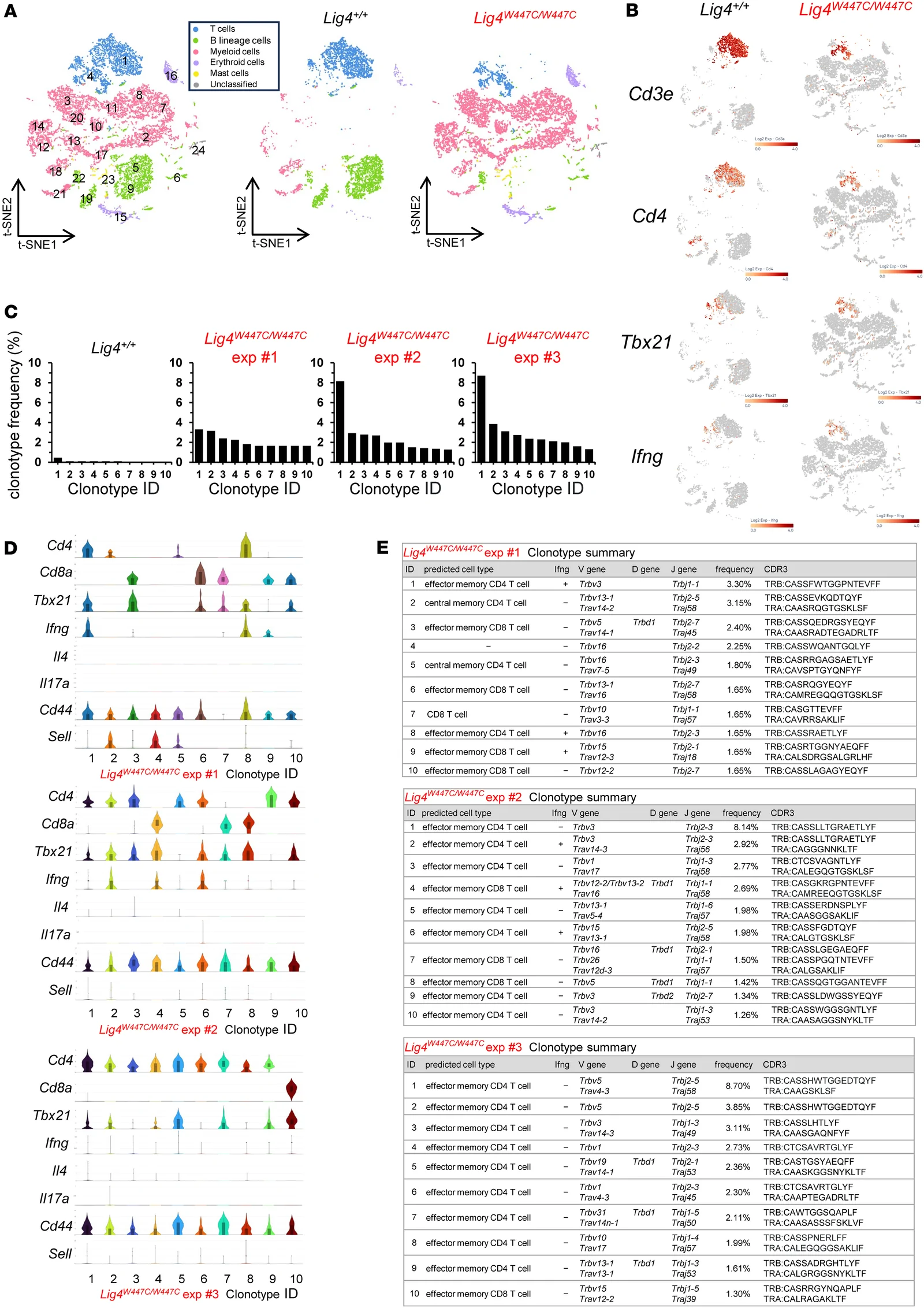
Single-cell RNA-seq of splenocyte samples. (**A**) The t-distributed stochastic neighbor embedding (t-SNE) plots of splenocytes from *Lig4^+/+^* and *Lig4^W447C/W447C^* mice based on 17,377 cells (*Lig4^+/+^*, 5,289 cells; *Lig4^W447C/W447C^*, 12,088 cells). Splenocytes were classified into 24 clusters. t-SNE plots show the data of total (left), *Lig4^+/+^* (middle), and *Lig4^W447C/W447C^* (right). Cells are grouped into 6 major cell populations: T cells, B-lineage cells, myeloid cells, erythroid cells, mast cells, and unclassified cells. A detailed cluster annotation is shown in Supplemental Figure 11. (**B**) Feature plots of *Cd3e*, *Cd4*, *Tbx21*, and *Ifng*. The color bar, from yellow to brown, reveals gradual expression intensity differences from low to high. (**C**) The clonotype frequencies of splenocytes from *Lig4^+/+^* mice and *Lig4^W447C/W447C^* mice (*n* = 3; experiments [exp] 1, 2, and 3). (**D**) Violin plots of the expression of the listed gene in the respective clonotype of each *Lig4^W447C/W447C^* mouse (*n* = 3; experiments 1, 2, and 3). (**E**) Clonotype summary of each *Lig4^W447C/W447C^* mouse (*n* = 3; experiments 1, 2, and 3).

**Figure 7:**
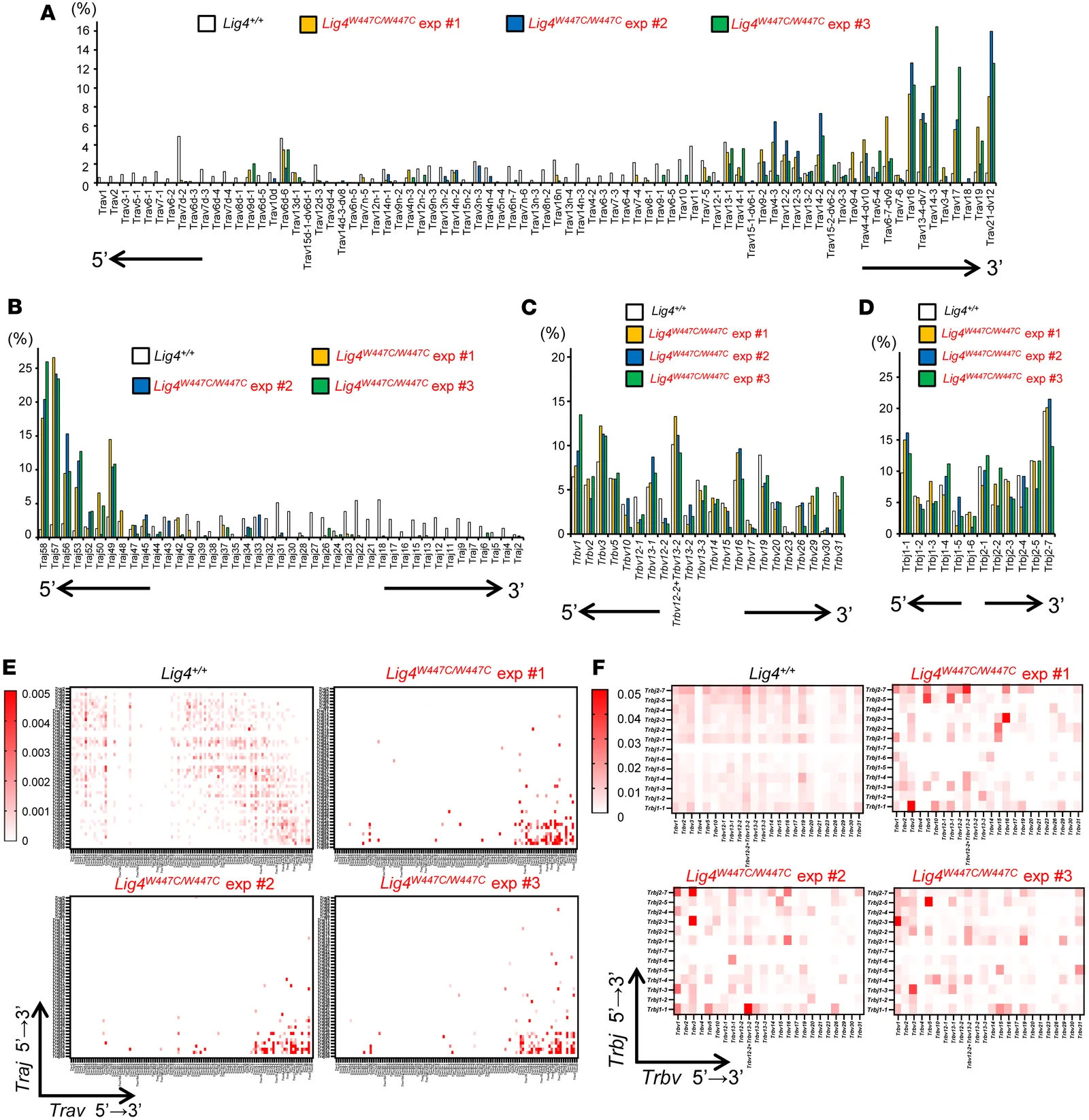
TCR repertoire analysis by single-cell RNA-seq of splenocyte samples. (**A**–**D**) The bar graphs depict the utilization frequencies of TCR gene segments in *Lig4^+/+^* mice (*n* = 1) and *Lig4^W447C/W447C^* mice (*n* = 3; experiments [exp] 1, 2, and 3). (**A**) *Trav* segments, arranged in chromosomal order from the 5′ end (left) to the 3′ end (right) of the *Trav* locus. (**B**) *Traj* segments, arranged in chromosomal order from the 5′ end (left) to the 3′ end (right) of the *Traj* locus. (**C**) *Trbv* segments, arranged in chromosomal order from the 5′ end (left) to the 3′ end (right) of the *Trbv* locus. (**D**) *Trbj* segments, arranged in chromosomal order from the 5′ end (left) to the 3′ end (right) of the *Trbj* locus. (**E** and **F**) Heatmaps showing the usage of *Trav*-*Traj* (**E**) and *Trbv*-*Trbj* (**F**) combinations. Splenocytes were prepared from individual mice (*Lig4^+/+^*, *n* = 1; *Lig4^W447C/W447C^*, *n* = 3; experiments 1, 2, and 3). (**E**) The *x* and *y* axes represent *Trav* and *Traj* segments, respectively, arranged from 5′ to 3′ end of the locus. The color gradient ranges from red to white, indicating high to low frequency of each *Trav*-*Traj* pair, respectively. The scale is shown on the right. (**F**) The *x* and *y* axes show *Trbv* and *Trbj* segments, respectively, arranged from 5′ to 3′ end of the locus. The same color gradient as shown in **E** is used.

## Version 1 09/01/2026

In-Press Preview

## Version 2 10/22/2026

Electronic publication
