# Supplementary material for: Hypomorphic Lig4 gene mutation in mice predisposes to Th1-skewed intestinal inflammation
Source: JCI Insight. 2026 Sep 1;11(20):e199040. doi: 10.1172/jci.insight.199040 (PMC13726569; doi:10.1172/jci.insight.199040)
Supplement: Supplementary file 1 [file jciinsight-11-199040-s074.pdf]

Supplemental Materials

Figures

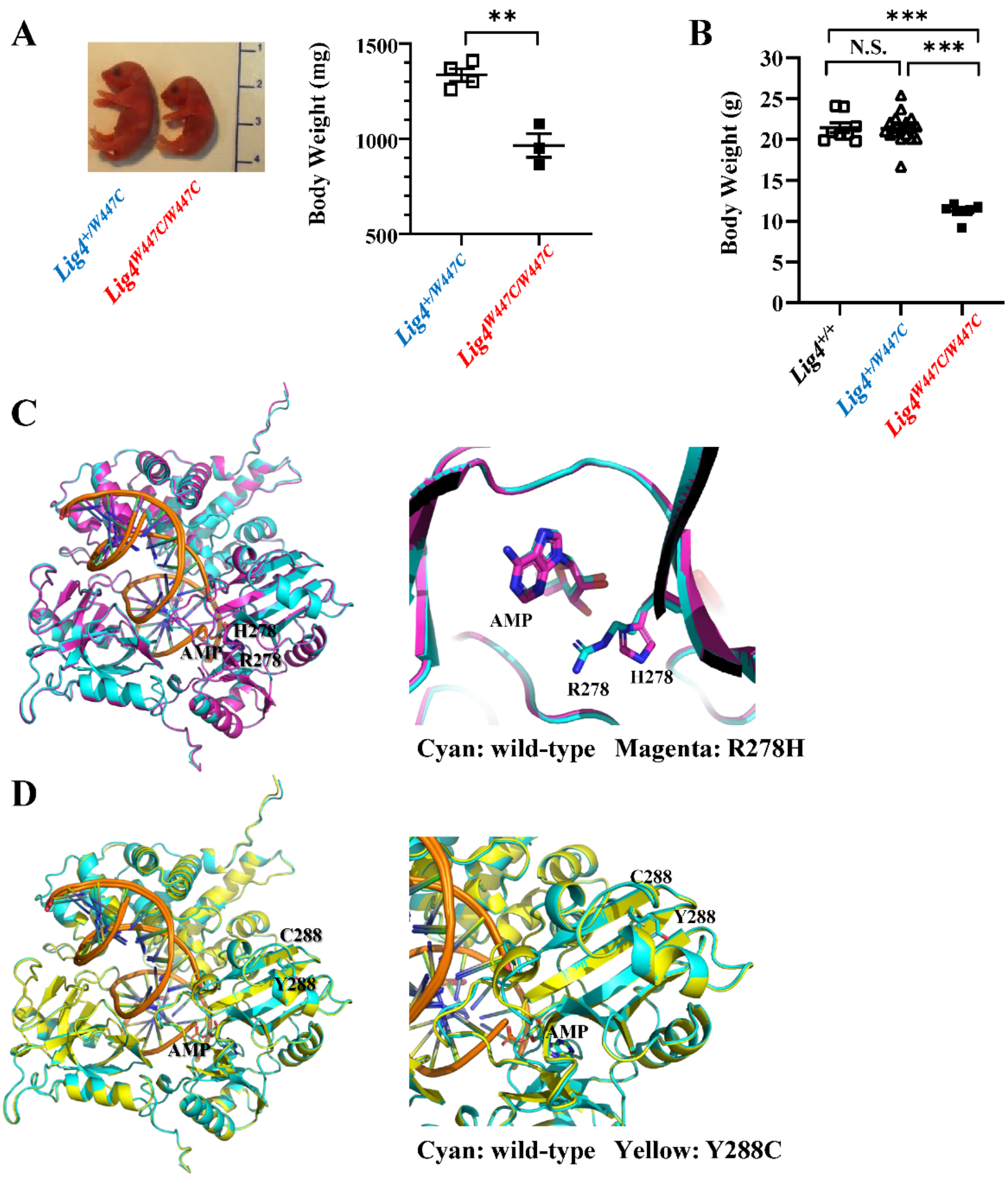

Figure S1. Related to Figure 1. Appearance and body weight of newborn mice, body weight of 6-week-old male mice, and structural prediction of LIGIV protein.

(**A**) The appearance of one-day-old *Lig4*$^{+/W447C}$ mice and *Lig4*$^{W447C/W447C}$ littermates. The number indicates units of centimeters. The graph (right) depicts the weight of one-day-old littermate mice. (*Lig4*$^{+/W447C}$ [n = 4] and *Lig4*$^{W447C/W447C}$ [n = 3]) (**B**) The graph depicts the body weight of six-week-

old male mice (*Lig4*$^{+/+}$ [n = 8], *Lig4*$^{+/W447C}$ [n = 17] and *Lig4*$^{W447C/W447C}$ [n = 6]). P-values were calculated using one-way analysis of variance (ANOVA) followed by Tukey's post hoc multiple comparison test. (**C** and **D**) Structural insights into R278H (**C**) and Y288C (**D**) mutations. (**C**) R278 (LIGIV wild-type) indicated cyan ribbon and LIGIV H278 did magenta ribbon. (**D**) Y288 (LIGIV wild type) indicated cyan ribbon and LIGIV C288 indicated yellow ribbon. (**C** and **D**, left panels) Neither mutation caused overall structural change. (**C**, right panel) R278 and H278 binding to AMP are shown in stick representation (cyan and magenta, respectively). R278H mutation decreased the interface area of AMP-binding. (**D**, right panel) The positions of Y288 (wild-type, cyan) and C288 (mutant, yellow) are shown relative to AMP. Neither residue directly contacted AMP.

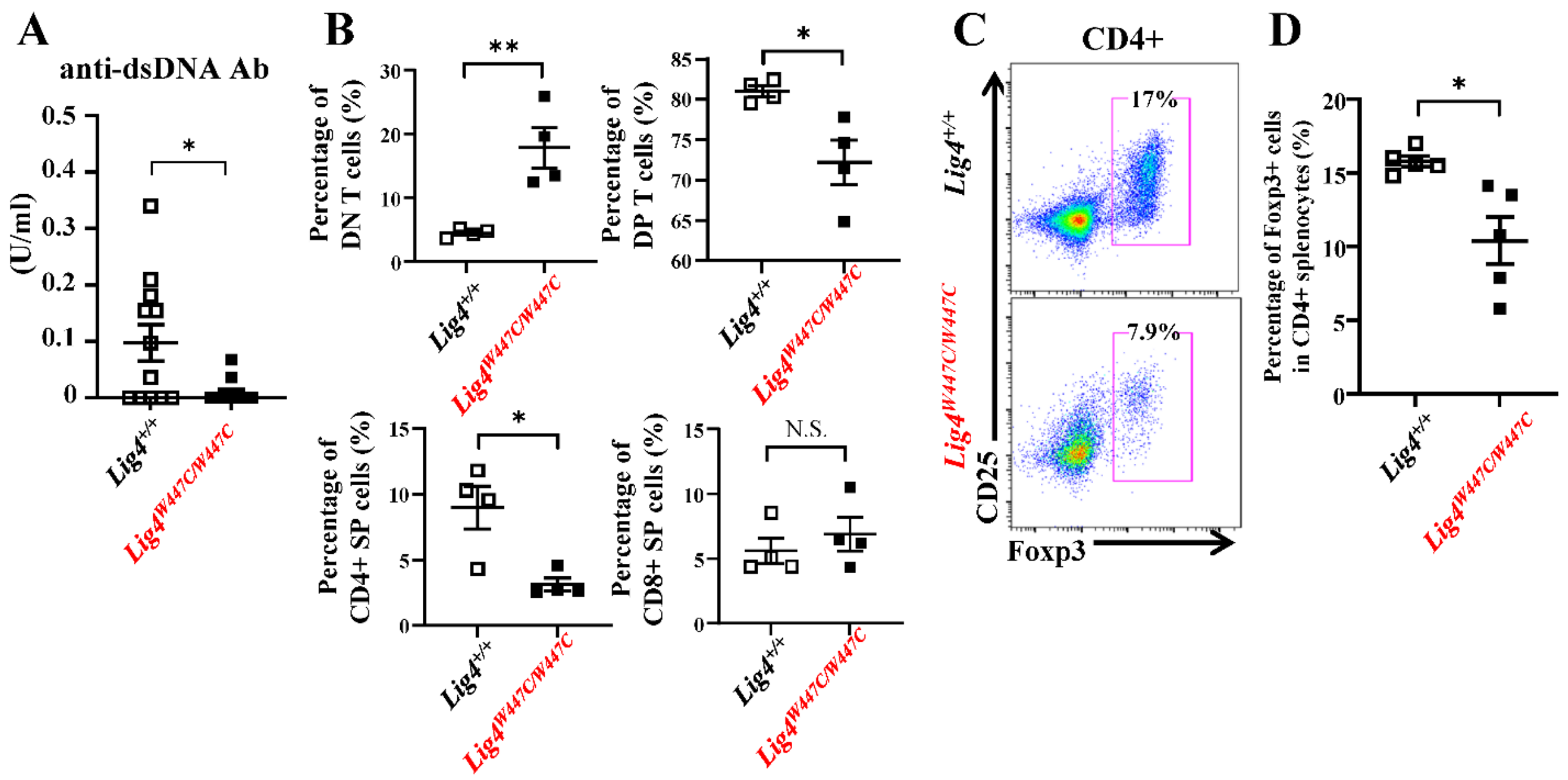

Figure S2. Related to Figure 2. Serum anti-dsDNA antibody levels and FACS analysis of thymic T cells and splenic regulatory T cells.

(**A**) Serum anti-dsDNA antibody levels in *Lig4*$^{+/+}$ and *Lig4*$^{W447C/W447C}$ mice, measured by ELISA (*Lig4*$^{+/+}$ [n = 12] and *Lig4*$^{W447C/W447C}$ [n = 12]; 9–23 weeks). (**B**) The percentages dynamics of thymic T cells at various developmental stages, including double negative (DN), double positive (DP), and single positive (SP) in *Lig4*$^{+/+}$ and *Lig4*$^{W447C/W447C}$ mice (*Lig4*$^{+/+}$ [n = 4] and *Lig4*$^{W447C/W447C}$ [n = 4];

7–16 weeks). (**C**) FACS analysis of splenocytes labeled with CD4, CD25 and Foxp3 antibodies from *Lig4*$^{+/+}$ and *Lig4*$^{W447C/W447C}$ mice. (**D**) Percentage of regulatory T cells in CD4+ splenocytes of *Lig4*$^{+/+}$ and *Lig4*$^{W447C/W447C}$ mice (*Lig4*$^{+/+}$ [*n* = 5] and *Lig4*$^{W447C/W447C}$ [*n* = 5]; 18–24 weeks) using FACS analysis.

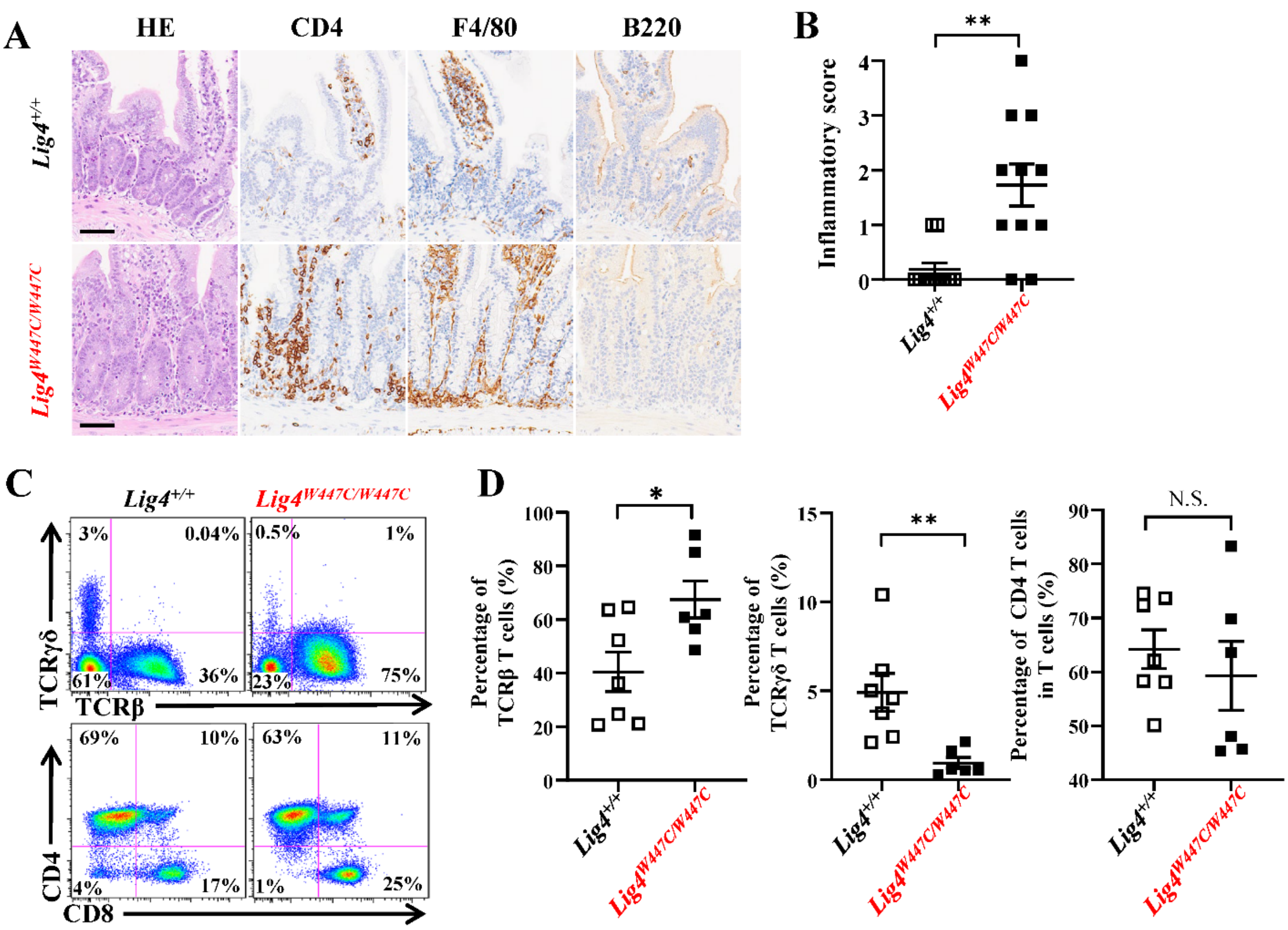

Figure S3. Related to Figure 3. Inflammation of the small intestine in *Lig4*$^{W447C/W447C}$ mice.

(**A**) H&E staining and immunohistochemical staining with CD4, F4/80 and B220 antibodies of the small intestine from *Lig4*$^{+/+}$ and *Lig4*$^{W447C/W447C}$ mice. Scale bar represents 60 µm. (**B**) A small intestine inflammatory score of each mouse (*Lig4*$^{+/+}$ [*n* = 11] and *Lig4*$^{W447C/W447C}$ [*n* = 11]; 7–18 weeks). (**C**) FACS analysis of T cells labeled with T-cell receptor (TCR)β, TCRγδ, CD4, and CD8 antibodies in the lamina propria of *Lig4*$^{+/+}$ and *Lig4*$^{W447C/W447C}$ mice. (**D**) The percentages of TCRβ^{+}, TCRγδ^{+}, and CD4^{+} T cells in the lamina propria of each mouse (*Lig4*$^{+/+}$ [*n* = 7] and *Lig4*$^{W447C/W447C}$ [*n* = 6]; 9–12

weeks). The data are presented as the mean ± SEM. *P*-values were calculated using the unpaired student's *t*-test.

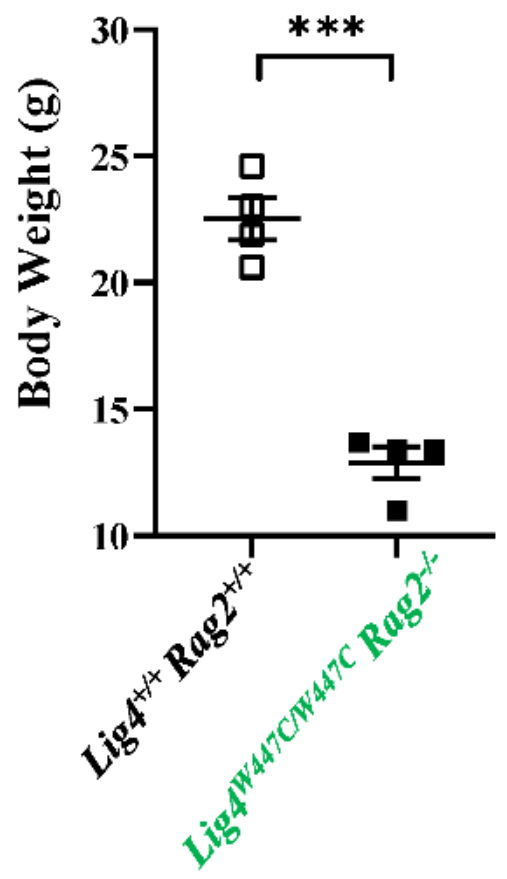

Figure S4. Related to Figure 3. Body weight of male mice.

The graph depicts the body weight of male mice (*Lig4*$^{+/+}$*Rag2*$^{+/+}$ [n = 4] and *Lig4*$^{\mathrm{W447C/W447C}}$*Rag2*$^{-/-}$ [n = 4]; 8 weeks). The data are presented as the mean ± SEM.

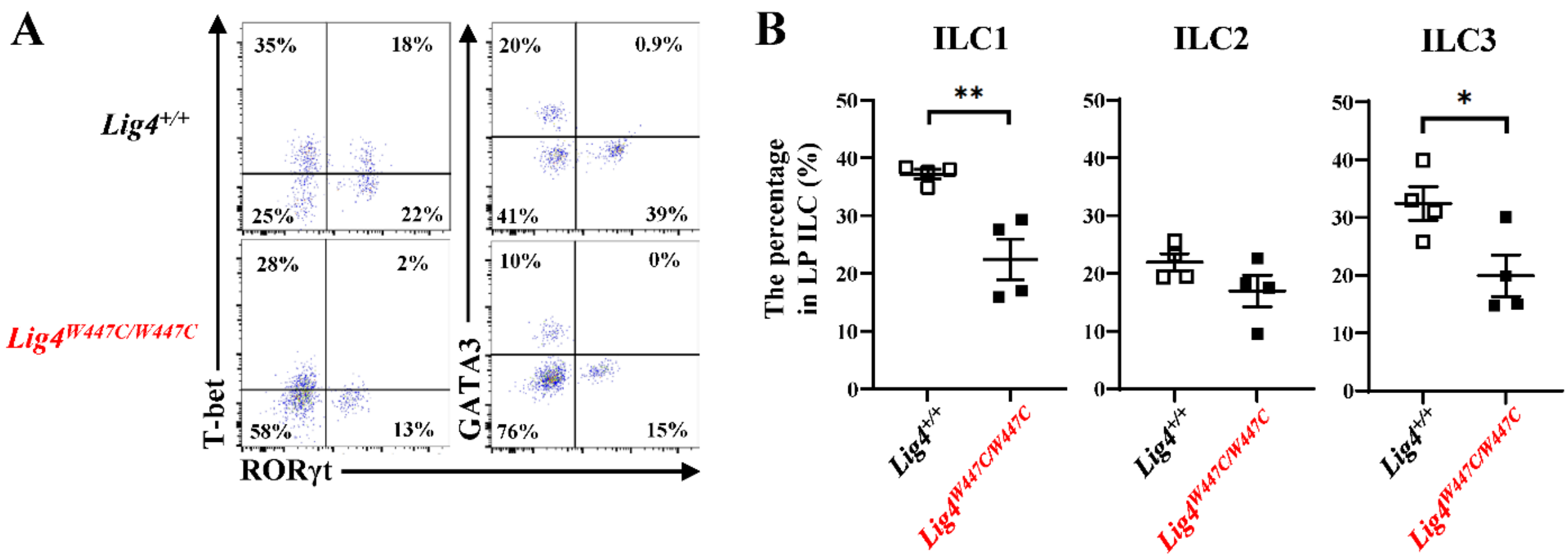

Figure S5. FACS analysis of innate lymphoid cells (ILCs).

(**A**) Dot plots of LP ILCs (LiveDead^{-}CD45.2^{+}(CD3e/CD19)$^{-}$Thy1.2^{+}). (**B**) Percentage of ILC1 (T-bet^{+}RORγt^{-}), ILC2 (GATA3^{+}), and ILC3 (RORγt^{+}) in LP ILC (*Lig4*$^{+/+}$ [n = 4] and *Lig4*$^{W447C/W447C}$ [n = 4]; 7–8 weeks).

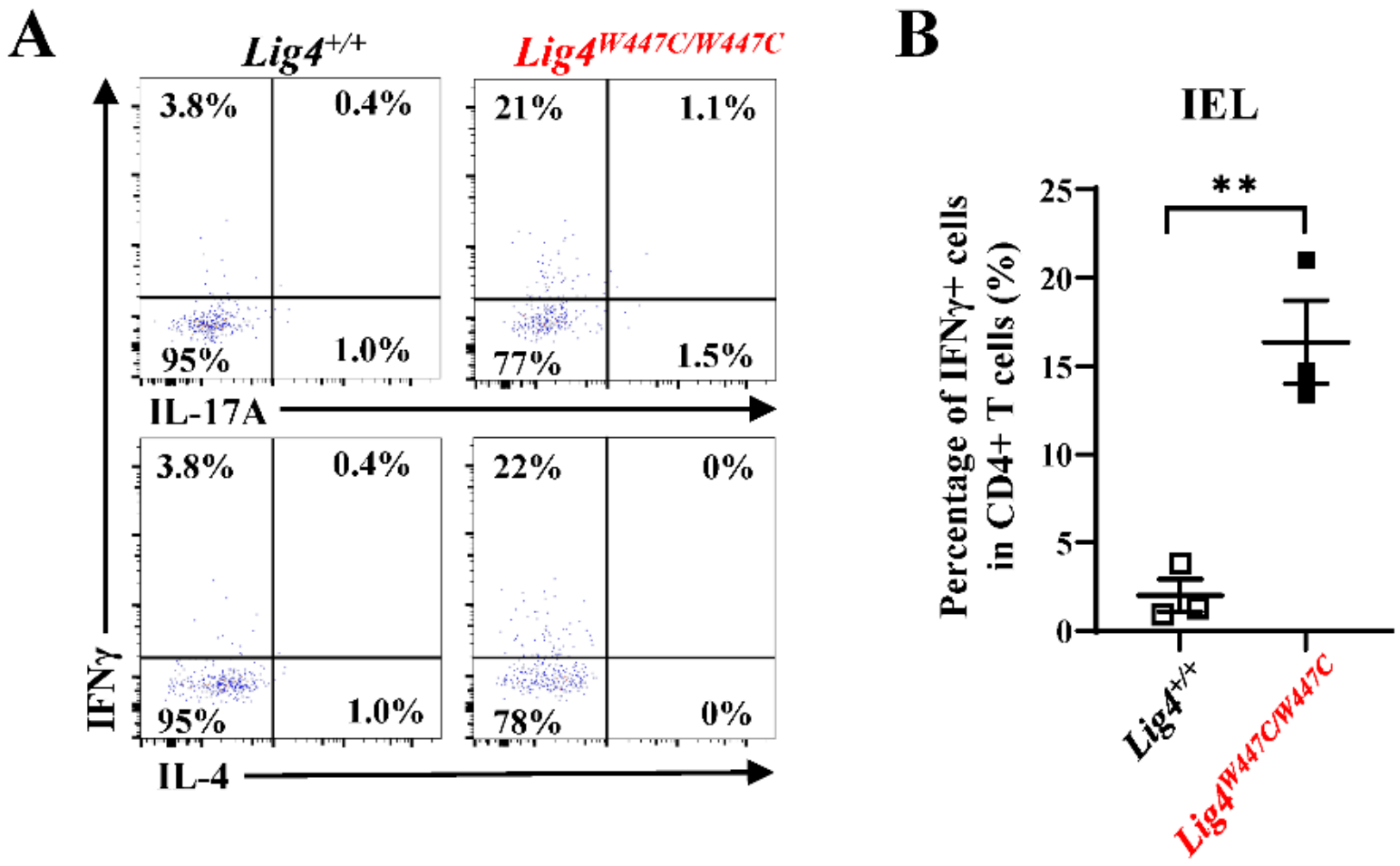

Figure S6. Related to Figure 5. FACS analysis of cytokine-producing $CD4^+$ T cells in intestinal IELs from *Lig4*$^{+/+}$ and *Lig4*$^{W447C/W447C}$.

(**A**) Intracellular cytokine staining (IFN-γ, IL-17A, and IL-4) of $CD4^+$ T cells in intestinal IELs from *Lig4*$^{+/+}$ and *Lig4*$^{W447C/W447C}$. (**B**) Percentage of IFN-γ^+ $CD4^+$ T cells in intestinal IELs (*Lig4*$^{+/+}$ [n = 3] and *Lig4*$^{W447C/W447C}$ [n = 3]; 6–15 weeks).

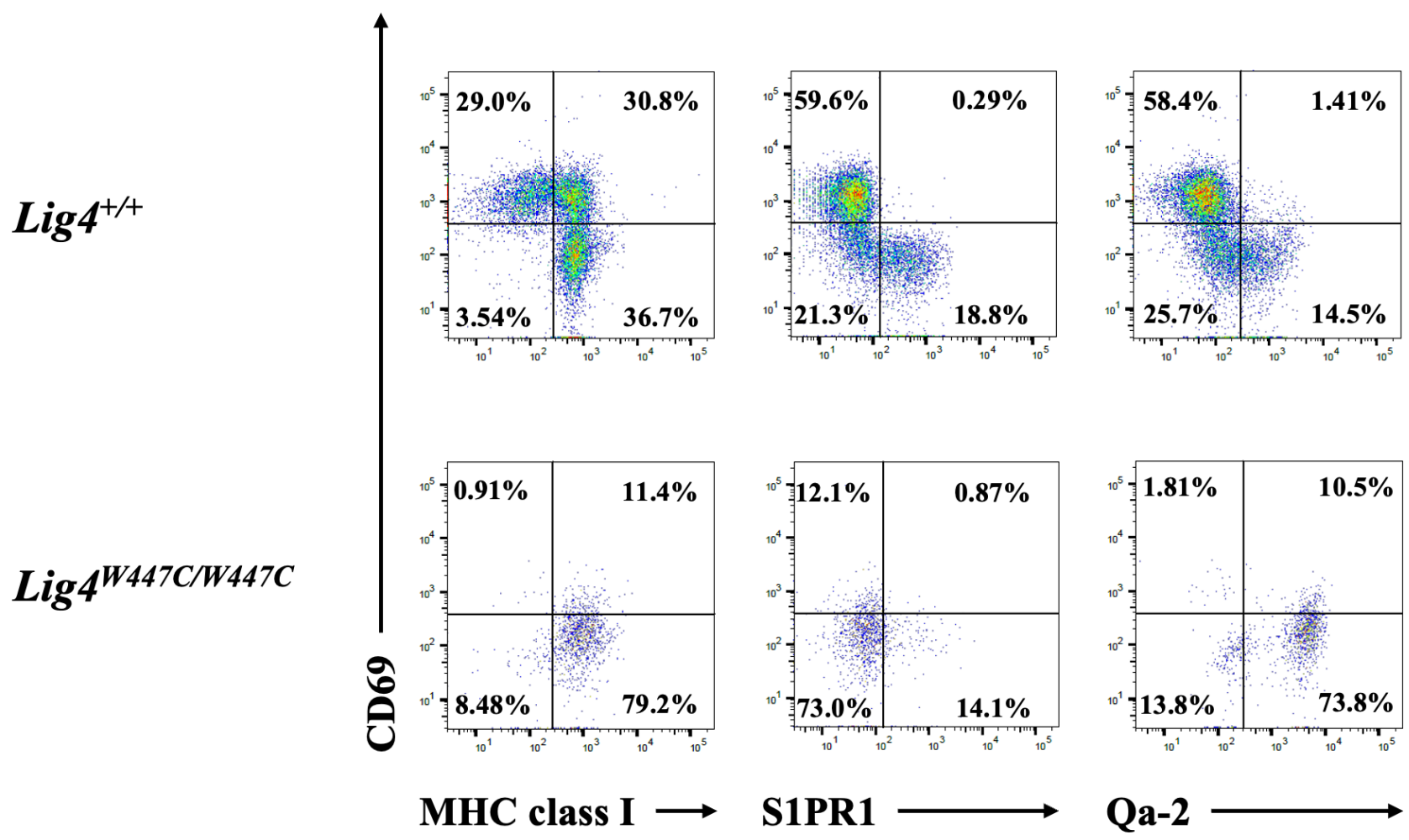

Figure S7. Flow cytometric analysis of CD4SP thymocytes

Thymocytes from four *Lig4*$^{+/+}$ and four *Lig4*$^{W447C/W447C}$ mice were analyzed by flow cytometry. Representative dot plots of CD4SP thymocytes from one mouse in each group are shown. Numbers in each quadrant indicate the percentage of cells within the CD4SP population.

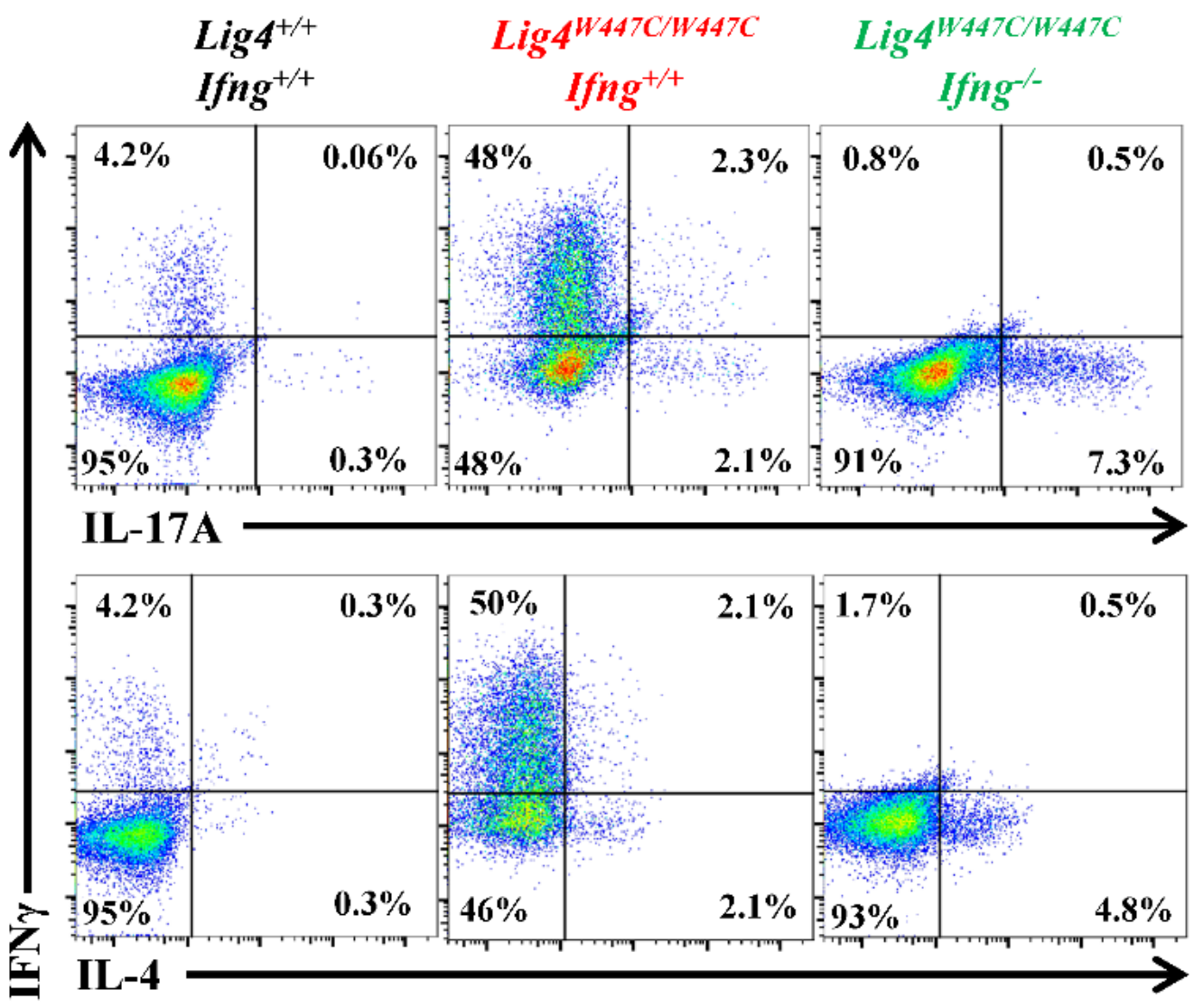

Figure S8. Related to Figure 5. Intracellular cytokine staining.

Intracellular cytokine staining (IFN-γ, IL-17A, and IL-4) of $CD4^+$ T cells in splenocytes from *Lig4*$^{+/+}$ *Ifng*$^{+/+}$, *Lig4*$^{W447C/W447C}$*Ifng*$^{+/+}$ and *Lig4*$^{W447C/W447C}$*Ifng*$^{-/-}$ mice.

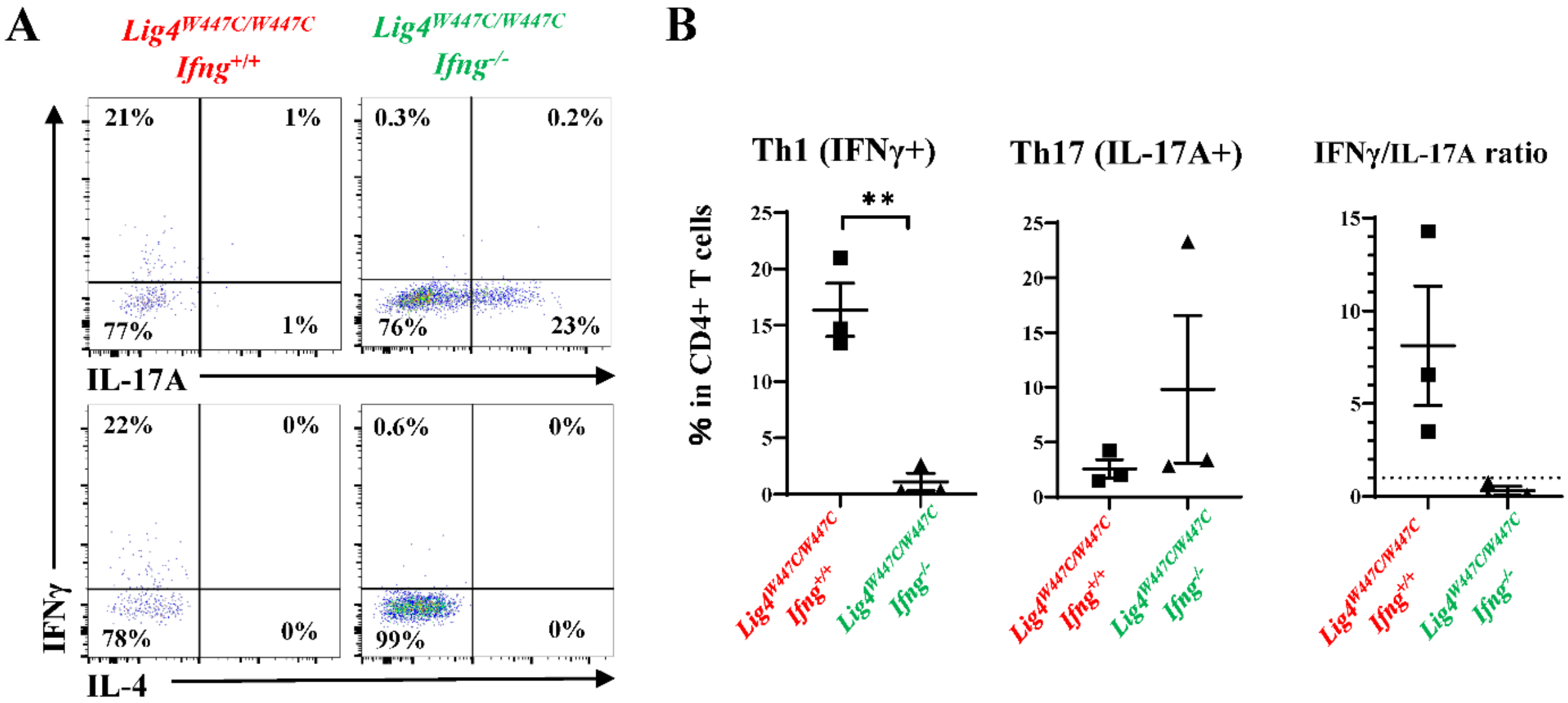

Figure S9. Related to Figure 5. FACS analysis of cytokine-producing $CD4^+$ T cells in intestinal IELs from *Lig4*$^{W447C/W447C}$ *Ifng*$^{+/+}$ and *Lig4*$^{W447C/W447C}$ *Ifng*$^{-/-}$.

(**A**) Intracellular cytokine staining (IFN-γ, IL-17A, and IL-4) of $CD4^+$ T cells in intestinal IELs from *Lig4*$^{W447C/W447C}$ *Ifng*$^{+/+}$ and *Lig4*$^{W447C/W447C}$ *Ifng*$^{-/-}$. (**B**) Percentage of IFN-γ^+ $CD4^+$ T cells in intestinal

IELs and their ratio (*Lig4*$^{W447C/W447C}$ *Ifng*$^{+/+}$ [n = 3] and *Lig4*$^{W447C/W447C}$ *Ifng*$^{-/-}$ [n = 3]; 6–11 weeks).

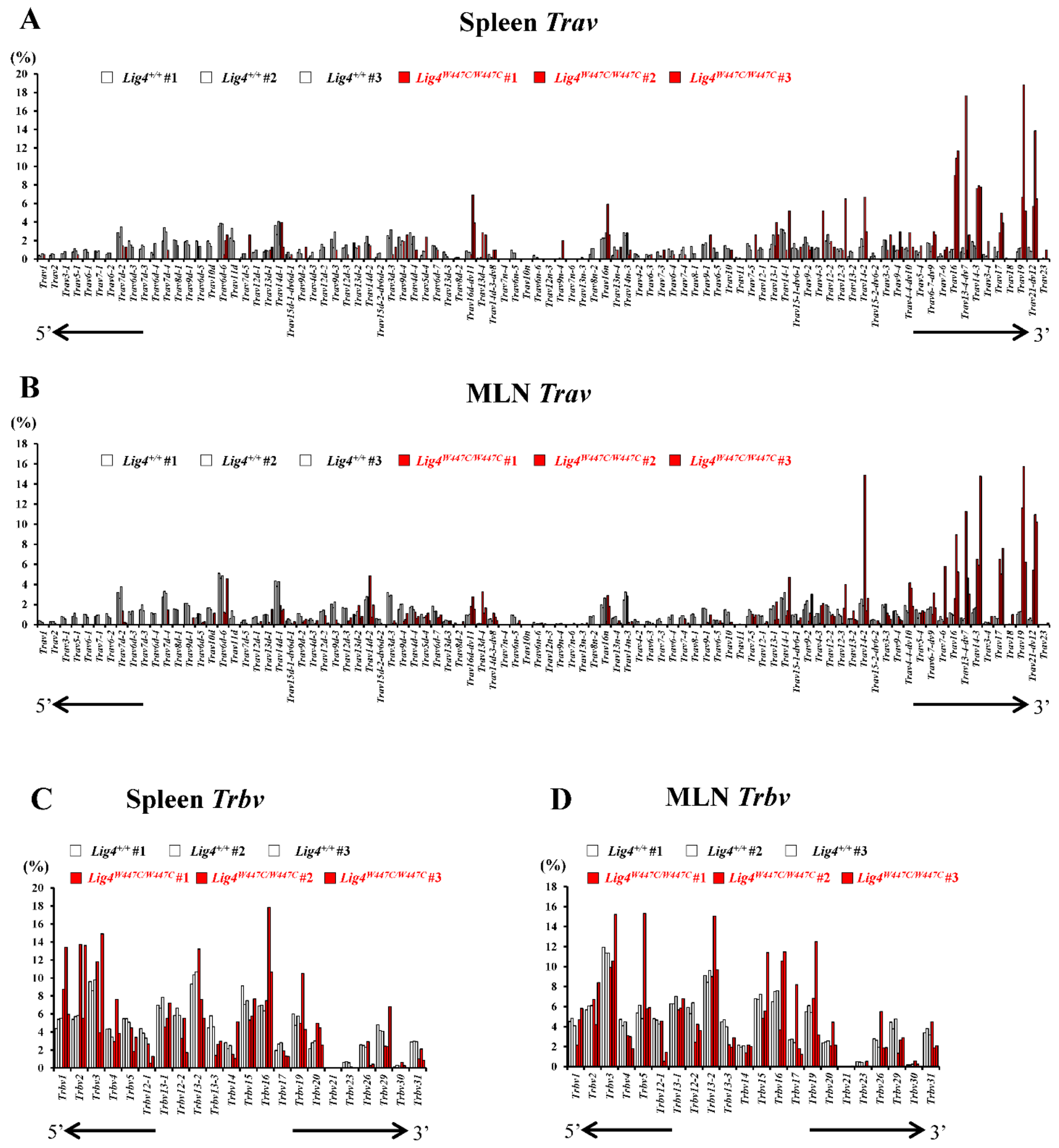

Figure S10. TCR repertoire analysis using bulk RNA-sequencing.

(**A-D**) The bar graphs show the relative expression levels of each *Trav* and *Trbv* gene segment analyzed by bulk RNA-sequencing. Data are presented as a percentage of the total *Trav* and *Trbv* gene expression in individual *Lig4*$^{+/+}$ mice [n=3; White bars] and *Lig4*$^{W447C/W447C}$ mice [n=3; red bars]. Gene

segments are arranged in chromosomal order, from the 5' end (left) to the 3' end (right) of the *Trav* and *Trbv* loci. (**A**) *Trav* expression in the spleen. (**B**) *Trav* expression in the MLNs. (**C**) *Trbv* expression in the spleen. (**D**) *Trbv* expression in the MLNs.

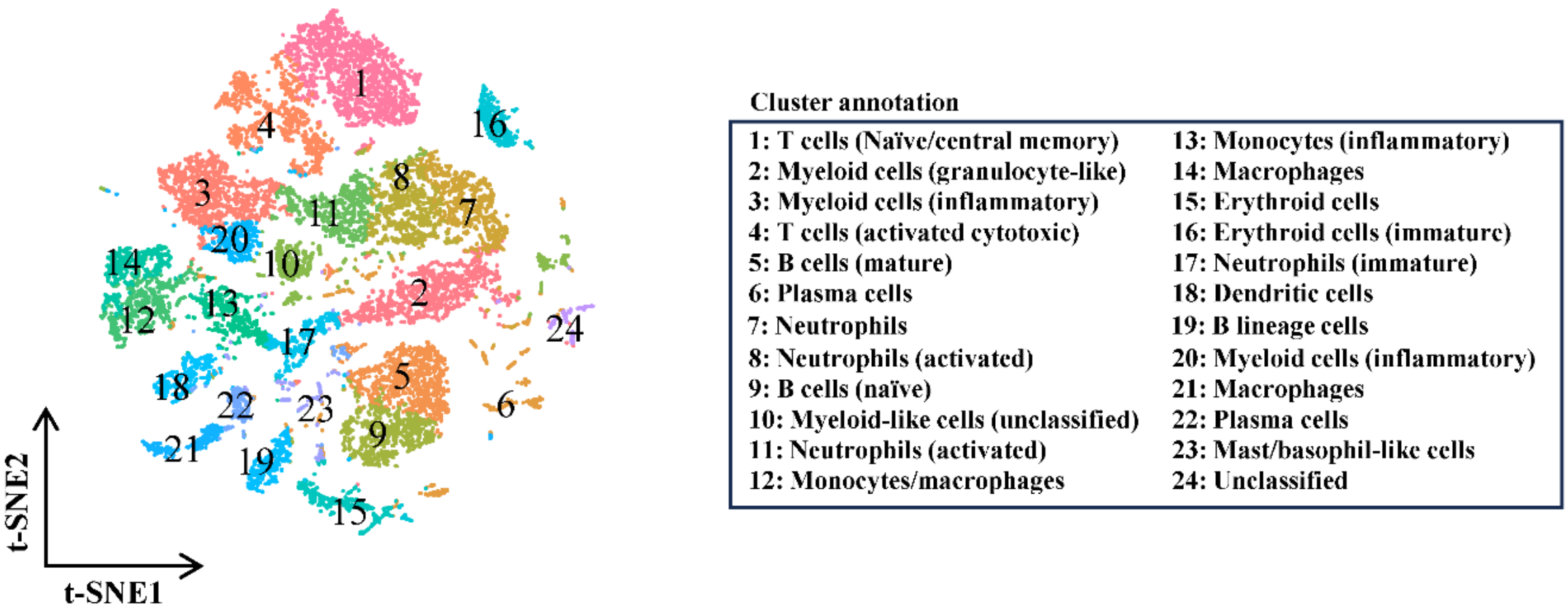

Figure S11. Related to Figure 6. Detailed cluster annotation of splenocyte single-cell RNA-seq data.

t-SNE plot of splenocytes from *Lig4*$^{+/+}$ and *Lig4*$^{W447C/W447C}$ mice showing 24 clusters. Cluster numbers correspond to the cell-type annotations listed in the panel, which were assigned based on marker gene expression. Marker genes used for cluster annotation are listed in Supplemental Table 5.

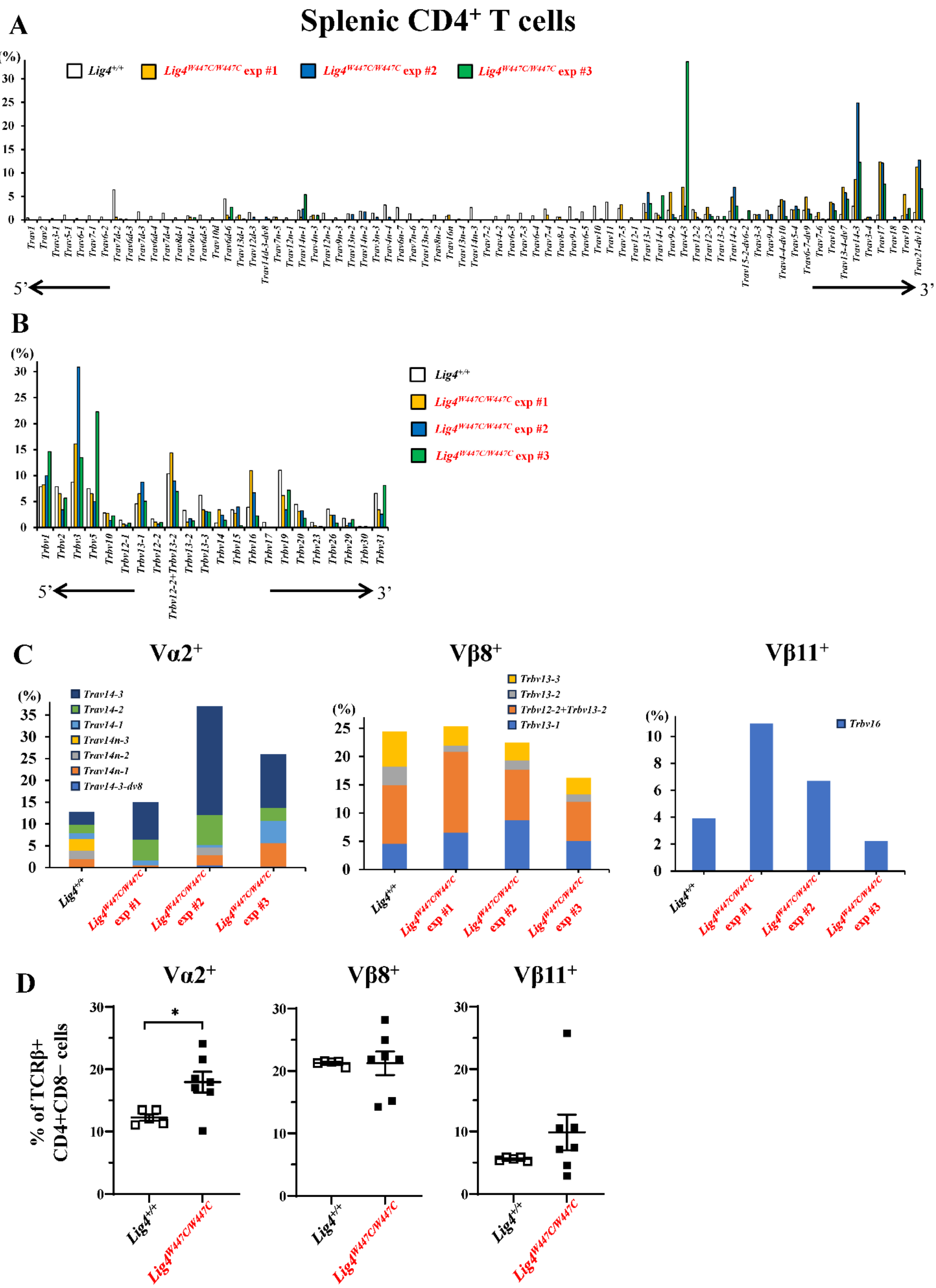
A
Splenic CD4+ T cells
(%)
Lig4+/+
Lig4W447C/W447C exp #1
Lig4W447C/W447C exp #2
Lig4W447C/W447C exp #3
5'
3'
B
(%)
Lig4+/+
Lig4W447C/W447C exp #1
Lig4W447C/W447C exp #2
Lig4W447C/W447C exp #3
Trbv1
Trbv2
Trbv3
Trbv5
Trbv10
Trbv12-1
Trbv13-1
Trbv12-2
Trbv12-2+Trbv13-2
Trbv13-2
Trbv13-3
Trbv14
Trbv15
Trbv16
Trbv17
Trbv19
Trbv20
Trbv23
Trbv26
Trbv29
Trbv30
Trbv31
5'
3'
C
Vα2+
Vβ8+
Vβ11+
(%)
Trav14-3
Trav14-2
Trav14-1
Trav14n-3
Trav14n-2
Trav14n-1
Trav14-3-dv8
Trbv13-3
Trbv13-2
Trbv12-2+Trbv13-2
Trbv13-1
Trbv16
Lig4+/+
Lig4W447C/W447C exp #1
Lig4W447C/W447C exp #2
Lig4W447C/W447C exp #3
D
Vα2+
Vβ8+
Vβ11+
% of TCRβ+ CD4+CD8− cells
*
Lig4+/+
Lig4W447C/W447C

Figure S12. Related to Figure 7. TCR repertoire analysis of splenic CD4^{+} T cells.

(**A**-**C**) TCR repertoire analysis by single-cell RNA-sequencing. Splenocytes expressing *Cd4* but not *Cd8α* were analyzed. The bar graphs depict the utilization frequencies of TCR gene segments in *Lig4*$^{+/+}$ mice [n = 1] and *Lig4*$^{W447C/W447C}$ mice [n = 3; exp #1, #2 and #3]. (**A**) *Trav* segments, arranged in chromosomal order from the 5' end (left) to the 3' end (right) of the *Trav* locus. (**B**) *Trbv* segments, arranged in chromosomal order from the 5' end (left) to the 3' end (right) of the *Trbv* locus. (**C**) Total frequencies of *Trav14* family (*Trav14-3, Trav14-2*, *Trav14-1*, *Trav14n-3*, *Trav14n-2*, *Trav14n-1*, *Trav14d-3-dv8*) (left), *Trbv13* family (*Trbv13-3, Trbv13-2*, *Trbv12-2+Trbv13-2*, *Trbv13-1*) (middle), *Trbv16* (right). (**D**) FACS TCR repertoire analysis of TCRβ^{+}CD4^{+}CD8^{-} splenocytes. Vα2 (left), Vβ8 (middle), Vβ11 (right) (*Lig4*$^{+/+}$ mice [n = 5] and *Lig4*$^{W447C/W447C}$ mice [n = 7]; 7–12 weeks). Data are presented as the mean ± SEM.

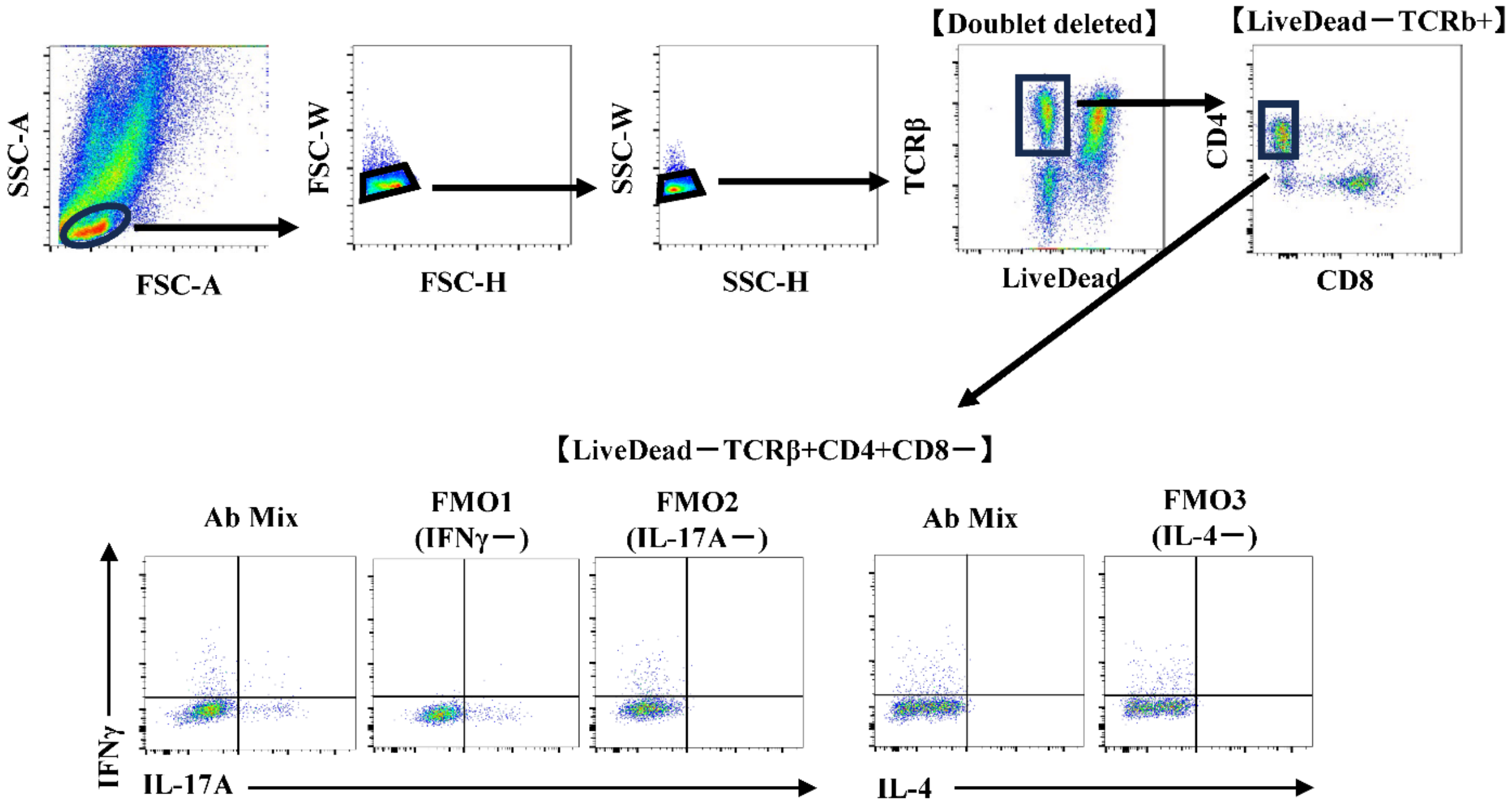

Figure S13. Representative gating strategy.

Representative flow cytometry dot plots showing the gating strategy used to identify cytokine-producing CD4^{+} T cells in LP. Fluorescence minus one (FMO) controls were used to define positive populations.

Supplemental Methods

Lig4$^{W447C/W447C}$ mice generation and genotyping

These *Lig4*$^{W447C/W447C}$ mice were generated using the CRISPR-Cas9 method (1). Briefly, guide RNA (gRNA; guide sequence: 5'-CAGACAAAAGAGGTGAAGGG-3') and Cas9 endonuclease mRNA were generated *in vitro* using the MEGAshortscript T7 (Life Technologies) and mMESSAGE mMACHINE T7 ULTRA (Life Technologies) transcription kits, respectively. The synthesized gRNA and Cas9 mRNA were purified using the MEGAclear kit (Life Technologies). Single-stranded oligodeoxynucleotides containing the *Lig4* c.1341G>T mutation (5'-CCCCCCACAATTAGGACGTCTAATTCATCCATTAGTCCACTGACGTACTCTGGTTTAATCTTTAGACACCCTTCACCTCTTTTGTCTGGCTTGTAAATGGACAGAGGGTGTTTAACCATGATCCCCTCTTC-3') were synthesized by Integrated DNA Technologies, Inc. To generate mutant mice, female B6C3F1 mice were super-ovulated and mated with C57BL/6N males (CLEA Japan Inc.). Fertilized one-cell-stage embryos were injected with gRNA, Cas9 mRNA, and single-stranded oligodeoxynucleotides. The c.1341G>T mutation was confirmed by Sanger sequencing, and the mutant mice were further backcrossed to C57BL/6N mice for more than six generations. We studied 8–16-week-old mutant mice and their littermates unless otherwise noted.

Genotyping was performed *via* polymerase chain reaction (PCR) amplification of tail DNAs using high-single nucleotide discrimination DNA polymerase (HiDi DNA polymerase; myPOLS Biotech GmbH). The following primers were used: control forward primer (CFP), 5'-AAGCCAGACAAAAGAGGTGAAGGGTGG-3'; mutation forward primer (MFP), 5'-AAGCCAGACAAAAGAGGTGAAGGGTGT-3'; and common reverse primer (CRP), 5'-TTTCATGGTGTAACCAGACCCAACACG-3'. PCR was performed with an initial denaturation at 95°C for 2 min, followed by 35 cycles of denaturation at 95°C for 15 seconds, annealing at 69°C for 10 seconds, extension at 72°C for 30 seconds, and then a final extension at 72°C for 1 min. The wild-

type and mutant alleles were detected as a 225 bp band using PCR with CFP-CRP and MFP-CRP primer pairs, respectively.

Generation of *Ifng*$^{-/-}$, *Lig4*$^{W447C/W447C}$*Rag2*$^{-/-}$, and *Lig4*$^{W447C/W447C}$ *Ifng*$^{-/-}$ mice

Ifng$^{-/-}$ mice were generated using the Alt-R CRISPR-Cas9 System (Integrated DNA Technologies, Inc.). CRISPR RNAs (crRNAs) including the target sequence, gRNA1 (Exon 2; 5'-GTCTCTTCTTGGATATCTGGAGG-3') or gRNA2 (between Exons 2 and 3; 5'-CATCAGCTGATAAAGCTAGGAGG-3') were designed, annealed with tracrRNA, and then incubated with Cas9 protein to generate the gRNA-Cas9 complex according to the manufacturer's instructions. The gRNA-Cas9 complex (the final 5 µM crRNA, 5 µM tracrRNA, and 1 µM Cas9) was injected into single-cell-stage fertilized eggs from C57BL/6N *via* electroporation (NEPA21 electroporator; Nepa Gene Co., Ltd). Approximately 350 injected eggs were transferred to the oviducts of pseudo-pregnant ICR females. After sequencing the PCR products of the targeted locus, six of the eight founders were found to be the *Ifng* variant. After that, one founder animal was selected to generate *Ifng* knockout mice.

Genotyping was performed *via* PCR amplification of tail DNAs using PrimeSTAR® Max DNA Polymerase (TaKaRa). The following primers were used: forward primer, 5'-CTACGGTCAATCCTCTCCTCAC-3'; and reverse primer, 5'-TTTGGATTCTCACGGCCATAC-3'. PCR was performed with an initial denaturation at 98°C for 5 min, followed by 35 cycles of denaturation at 98°C for 10 seconds, annealing at 68°C for 5 seconds, extension at 72°C for 1 min, and then a final extension at 72°C for 1 min. The wild type was detected as a 1,620 bp band, while mutant alleles were detected as a 1224 bp band using PCR with a combination of primer pairs.

Heterozygous *Lig4*$^{W447C/+}$ mice were mated to *Rag2*$^{-/-}$ or *Ifng*$^{-/-}$ mice to generate *Lig4*$^{W447C/W447C}$ *Rag2*$^{-/-}$ and *Lig4*$^{W447C/W447C}$ *Ifng*$^{-/-}$ mice. C57BL/6N mice were used, and *Rag2* was genotyped using PCR, as previously described (2).

Preparation of MEFs

E14.5 embryos were removed, one by one, from the uterus, and the head-removed bodies were cut into small pieces with scissors. Then, they were cultured in a T-25 (25 cm^2) culture flask (Falcon; Corning Inc.) per embryo with 2 ml of aMEM (Nacalai Tesque) supplemented with 10% fetal bovine serum (Gibco; Thermo Fisher Scientific) at 37°C under humidified 5% CO_2 conditions. After two-day culture, the cells were fed with 2 ml of the medium, and then the medium was changed at another two-day culture. When growing cells became confluent, they were suspended in the medium supplemented with 10% dimethyl sulfoxide (Nacalai Tesque) and frozen in liquid nitrogen for stock. The frozen stock of MEFs were thawed and allowed to grow for subsequent experiments.

Measurement of serum anti-dsDNA antibodies levels in mice

Serum anti-dsDNA antibodies levels of $Lig4^{W447C/W447C}$ mice and wild-type C57BL/6N were measured by ELISA using LBIS Mouse anti-dsDNA ELISA Kit (Cat# 637-02691, FUJIFILM), following the manufacturer's instructions.

Histopathological evaluation of enteritis

Enteritis was assessed using the inflammatory score reported by Erben et al (3). The inflammation score ranged from 0 (no disease) to 5 (severe disease) and was determined based on the severity and extent of inflammatory cell infiltration, epithelial changes, and mucosal architecture.

Supplementary References

1. Kanazawa N, et al. Heterozygous missense variant of the proteasome subunit β-type 9 causes neonatal-onset autoinflammation and immunodeficiency. *Nat Commun*. 2021;12(1):6819.

2. Shinkai Y, et al. RAG-2-deficient mice lack mature lymphocytes owing to inability to initiate

V(D)J rearrangement. *Cell*. 1992;68(5):855-867.

3. Erben U, et al. A guide to histomorphological evaluation of intestinal inflammation in mouse models. *Int J Clin Exp Pathol*. 2014;7(8):4557-4576.
