# Supplementary material for: Hypomorphic Lig4 gene mutation in mice predisposes to Th1-skewed intestinal inflammation
Source: JCI Insight. 2026 Sep 1;11(20):e199040. doi: 10.1172/jci.insight.199040 (PMC13726569; doi:10.1172/jci.insight.199040)
Supplement: Supplementary file 2 [file jciinsight-11-199040-s075.pdf]

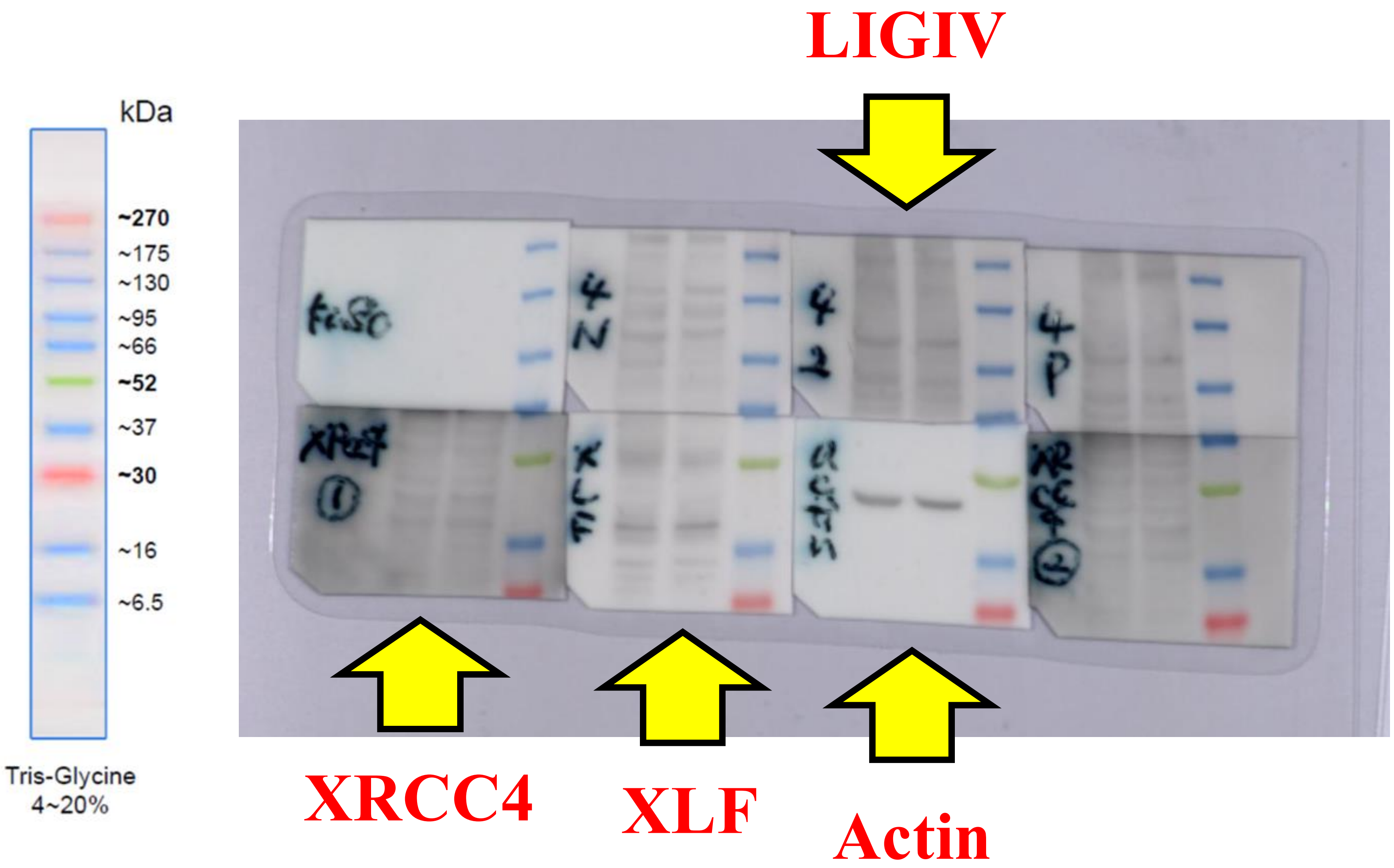

Figure 1C Western blots raw data
